# Lipolysis drives expression of the constitutively active receptor GPR3 to induce adipose thermogenesis

**DOI:** 10.1016/j.cell.2021.04.037

**Published:** 2021-06-24

**Authors:** Olivia Sveidahl Johansen, Tao Ma, Jakob Bondo Hansen, Lasse Kruse Markussen, Renate Schreiber, Laia Reverte-Salisa, Hua Dong, Dan Ploug Christensen, Wenfei Sun, Thorsten Gnad, Iuliia Karavaeva, Thomas Svava Nielsen, Sander Kooijman, Cheryl Cero, Oksana Dmytriyeva, Yachen Shen, Maria Razzoli, Shannon L. O’Brien, Eline N. Kuipers, Carsten Haagen Nielsen, William Orchard, Nienke Willemsen, Naja Zenius Jespersen, Morten Lundh, Elahu Gosney Sustarsic, Cecilie Mørch Hallgren, Mikkel Frost, Seth McGonigle, Marie Sophie Isidor, Christa Broholm, Oluf Pedersen, Jacob Bo Hansen, Niels Grarup, Torben Hansen, Andreas Kjær, James G. Granneman, M. Madan Babu, Davide Calebiro, Søren Nielsen, Mikael Rydén, Raymond Soccio, Patrick C.N. Rensen, Jonas Thue Treebak, Thue Walter Schwartz, Brice Emanuelli, Alessandro Bartolomucci, Alexander Pfeifer, Rudolf Zechner, Camilla Scheele, Susanne Mandrup, Zachary Gerhart-Hines

**Affiliations:** 1 Novo Nordisk Foundation Center for Basic Metabolic Research, University of Copenhagen, Copenhagen, Denmark; 2 Embark Biotech ApS, Copenhagen, Denmark; 3 Center for Adipocyte Signaling, University of Southern Denmark, Odense, Denmark; 4 Functional Genomics and Metabolism Research Unit, Department of Biochemistry and Molecular Biology, University of Southern Denmark, Odense, Denmark; 5 Institute of Molecular Biosciences, University of Graz, Graz, Austria; 6 Institute of Pharmacology and Toxicology, University Hospital, University of Bonn, Bonn, Germany; 7 Institute of Food, Nutrition and Health, ETH Zurich, Zurich, Switzerland; 8 Department of Medicine, Division of Endocrinology, Leiden University Medical Center, Leiden, the Netherlands; 9 Einthoven Laboratory for Experimental Vascular Medicine, Leiden University Medical Center, Leiden, the Netherlands; 10 Department of Integrative Biology and Physiology, University of Minnesota, Minneapolis, MN, USA; 11 Institute for Diabetes, Obesity, and Metabolism, Department of Medicine, Division of Endocrinology, Diabetes, and Metabolism, University of Pennsylvania Perelman School of Medicine, Philadelphia, PA, USA; 12 Institute of Metabolism and Systems Research, University of Birmingham, Birmingham, UK; 13 Centre of Membrane Proteins and Receptors (COMPARE), Universities of Birmingham and Nottingham, Birmingham, UK; 14 Institute of Pharmacology and Toxicology and Bio-Imaging Center, University of Würzburg, Würzburg, Germany; 15 Department of Biomedical Sciences, University of Copenhagen, Copenhagen, Denmark; 16 Department of Clinical Physiology, Nuclear Medicine & PET and Cluster for Molecular Imaging, Rigshospitalet, Copenhagen, Denmark; 17 MRC Laboratory of Molecular Biology, Cambridge, UK; 18 Department of Structural Biology and Center for Data Driven Discovery, St. Jude Children’s Research Hospital, Memphis, TN, USA; 19 Section for Cell Biology and Physiology, Department of Biology, University of Copenhagen, Copenhagen, Denmark; 20 Center for Molecular Medicine and Genetics, Wayne State University School of Medicine, Detroit, MI, USA; 21 Department of Medicine (H7), Karolinska Institute, Karolinska University Hospital, Stockholm, Sweden; 22 Centre of Inflammation and Metabolism and Centre for Physical Activity Research, Rigshospitalet, University Hospital of Copenhagen, Copenhagen, Denmark; 23 BioTechMed-Graz, Graz, Austria

**Keywords:** G protein-coupled receptor, GPCR, GPR3, brown adipose tissue, thermogenesis, lipolysis, constitutively active, transcription, adrenergic receptor, energy expenditure

## Abstract

Thermogenic adipocytes possess a therapeutically appealing, energy-expending capacity, which is canonically cold-induced by ligand-dependent activation of β-adrenergic G protein-coupled receptors (GPCRs). Here, we uncover an alternate paradigm of GPCR-mediated adipose thermogenesis through the constitutively active receptor, GPR3. We show that the N terminus of GPR3 confers intrinsic signaling activity, resulting in continuous Gs-coupling and cAMP production without an exogenous ligand. Thus, transcriptional induction of *Gpr3* represents the regulatory parallel to ligand-binding of conventional GPCRs. Consequently, increasing *Gpr3* expression in thermogenic adipocytes is alone sufficient to drive energy expenditure and counteract metabolic disease in mice. *Gpr3* transcription is cold-stimulated by a lipolytic signal, and dietary fat potentiates GPR3-dependent thermogenesis to amplify the response to caloric excess. Moreover, we find GPR3 to be an essential, adrenergic-independent regulator of human brown adipocytes. Taken together, our findings reveal a noncanonical mechanism of GPCR control and thermogenic activation through the lipolysis-induced expression of constitutively active GPR3.

## Highlights

• *Gpr3* is a cold-induced Gs-coupled receptor in brown and beige adipose tissue
• A noncanonical lipolytic signal triggers *Gpr3* transcription during cold exposure
• GPR3 is a nonadrenergic activator of mouse and human thermogenic adipocytes
• GPR3 drives thermogenesis without a ligand via its intrinsic Gs-coupling activity

## Graphical abstract

**Figure:**
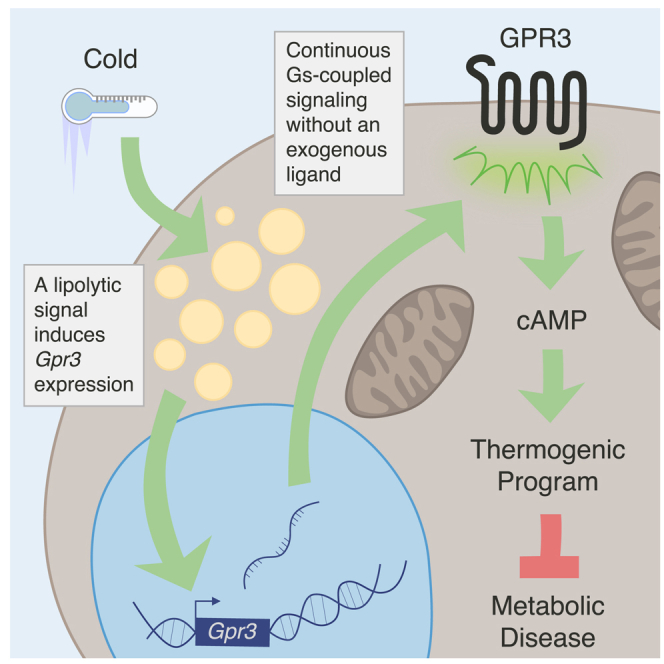

## Teaser

Cold-induced lipolysis drives the expression of a constitutively active GPCR that regulates thermogenesis in mouse and human adipocytes independent of sympathetic or adrenergic inputs.

## Introduction

Exposure to environmental cold stimulates thermogenic catabolism of lipids and carbohydrates in brown adipose tissue (BAT) (Cannon and Nedergaard, 2004; Rosen and Spiegelman, 2014). This physiological response improves metabolic homeostasis (Chondronikola et al., 2016; van der Lans et al., 2013; Sidossis and Kajimura, 2015) and is strongly influenced by G protein-coupled receptors (GPCRs) (Beaudry et al., 2019b, 2019a; Cannon and Nedergaard, 2004; Caron et al., 2019; Cero et al., 2016; Collins, 2012; Gnad et al., 2014; Klepac et al., 2016; Li et al., 2018; Lowell and Spiegelman, 2000). GPCRs are cell surface receptors with seven transmembrane domains that transduce signals through heterotrimeric complexes of G proteins, Gα, Gβ, and Gγ (Kobilka, 2007; Wettschureck and Offermanns, 2005). In the conventional model of GPCR activation, ligand binding triggers a conformational change in the receptor that causes “inactive” Gα proteins to exchange bound GDP for GTP (Kobilka, 2007; Wettschureck and Offermanns, 2005). Newly GTP-bound Gα proteins are liberated from the heterotrimeric complex and convey downstream signals.

Of the four primary Gα subtypes (Gs, Gi, Gq, and G12/13), BAT activation is predominantly ascribed to the Gs-coupled family, which signals through increased cyclic AMP (cAMP). This class is exemplified by the β-adrenergic receptors (ADRB1, ADRB2, and ADRB3), which represent the canonical means of sympathetic, ligand-mediated thermogenic control (Collins, 2012). β-adrenergic agonism potently stimulates adipose energy expenditure in mice (Cannon and Nedergaard, 2004; Collins, 2012) and humans (Blondin et al., 2020; Cypess et al., 2015) and genetic deletion of ADRBs in mice results in impaired acute cold and stress-induced thermogenesis (Bachman et al., 2002; Razzoli et al., 2015). Several additional Gs-coupled receptors have been shown to activate adipose thermogenesis including receptors for secretin (Li et al., 2018), glucagon (Beaudry et al., 2019a), glucose-dependent insulinotropic polypeptide (Beaudry et al., 2019b), adrenocorticotropic hormone (Schnabl et al., 2019), and adenosine (Gnad et al., 2014).

Gs-coupled cAMP production in brown adipocytes triggers a diverse array of downstream thermogenic events (Caron et al., 2019; Wang et al., 2019). Metabolic substrates are consumed (Bartelt et al., 2011; Cannon and Nedergaard, 2004; Mills et al., 2018) to fuel mitochondrial futile cycles (Kazak et al., 2015) and uncoupling protein 1 (UCP1)-dependent respiration (Golozoubova et al., 2001) to ultimately convert chemical energy to heat. Gs-signaling also re-wires transcriptional networks to support the increased catabolic demand (Harms and Seale, 2013) and stimulates the recruitment of thermogenically competent beige adipocytes in the subcutaneous adipose depots (Shabalina et al., 2013; Wu et al., 2012). Taken together, Gs-coupled signaling orchestrates both acute and adaptive phases of adipose thermogenesis.

**Figure 1:**
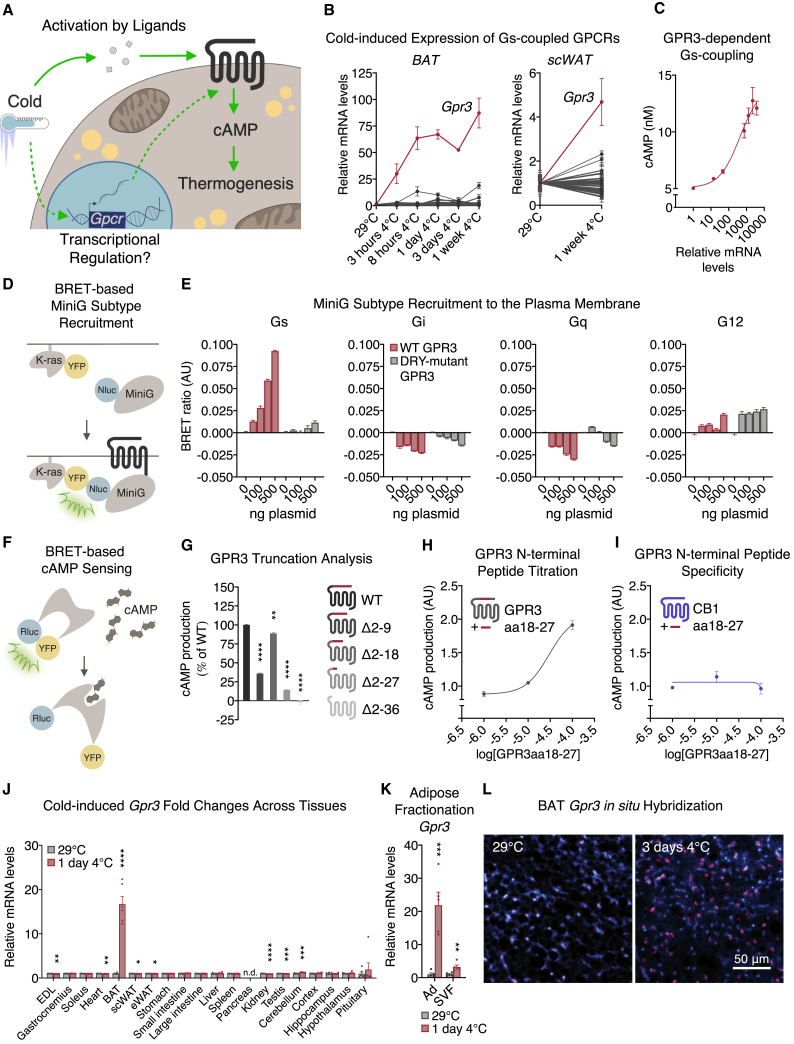
The constitutively active receptor GPR3 is the most cold-induced Gs-coupled GPCR in thermogenic adipose tissue (A) Schematic depicting canonical ligand-dependent (solid line) versus hypothesized transcriptional control (dotted line) of Gs-coupled receptors in thermogenic adipocytes. (B) Induction of Gs-coupled receptors in brown (left) and subcutaneous (right) white adipose depots during adaptation to cold. Statistical significance for each receptor at individual time points is indicated in Table S1 (BAT) and Table S2 (scWAT). (C) cAMP accumulation in COS-7 cells transfected with increasing concentrations of GPR3 plasmid; gene expression data presented in log scale. (D) Schematic depicting the bioluminescence resonance energy transfer (BRET) assay used to assess. (E) G protein recruitment to wild-type (WT) and DRY-mutant GPR3. (F) Scheme depicting the BRET assay used to assess. (G–I) (G) cAMP levels produced by WT and N-terminal truncations of GPR3 and cAMP production induced by N-terminal GPR3 fragment aa18-27 on (H) WT GPR3 and (I) cannabinoid 1 receptor (CB1). (J) Tissue panel of cold-induced fold changes in *Gpr3* expression. (K) Differential levels of cold-induced *Gpr3* expression in BAT adipocytes (Ad) and stromal vascular fraction (SVF). (L) *In situ* hybridization (ISH) of *Gpr3* mRNA (red) in BAT of thermoneutral-housed or cold-challenged mice. Nuclei in BAT are stained with DAPI (blue). For all panels, error bars represent ±SEM, p ≤ 0.05 = ∗, p ≤ 0.01 = ∗∗, p ≤ 0.001 = ∗∗∗, p ≤ 0.0001 = ∗∗∗∗, t test (K and J) or Bonferroni's multiple comparisons test (G). See also Figure S1.

Sympathetic nerve stimulation and pharmacological mimicry targeting β-adrenergic receptors have underscored the potential of Gs-induced adipose thermogenesis to counteract metabolic disease (Bachman et al., 2002; Collins, 2012; Xiao et al., 2015). Yet, the cardiovascular risks associated with the use of sympathomimetic drugs likely preclude β-adrenergic activation as a standalone clinical modality. Thus, uncovering additional regulatory events for Gs-coupled receptors that precipitate the thermogenic program is critical for developing new therapeutic strategies. Most focus has centered on the canonical point of control for GPCRs, which is the ligand-binding event that instigates all downstream cascades. Far less explored and understood is the extent to which receptor signaling is impacted by acute transcriptional control of the GPCRs themselves. Here, we set out to explore a potential regulatory paradigm whereby adipocytes acutely and dynamically modulate expression of GPCR genes during cold exposure to influence the thermogenic trajectory (Figure 1A). We found that a noncanonical lipolytic signal directly stimulates the transcription of G protein-coupled receptor 3 (*Gpr3*). We further show that the N terminus of GPR3 confers the innate ability to potently signal through Gs-coupling without the need of an exogenous ligand. Therefore, increasing *Gpr3* expression is fully sufficient to orchestrate cAMP-driven adipose thermogenesis. These findings represent a mode of GPCR control in which transcriptional induction of a receptor with intrinsic activity is analogous to ligand-binding activation of a conventional GPCR.

## Results

### The constitutively active receptor GPR3 is the most cold-induced Gs-coupled GPCR in thermogenic adipose tissue

**Figure S1:**
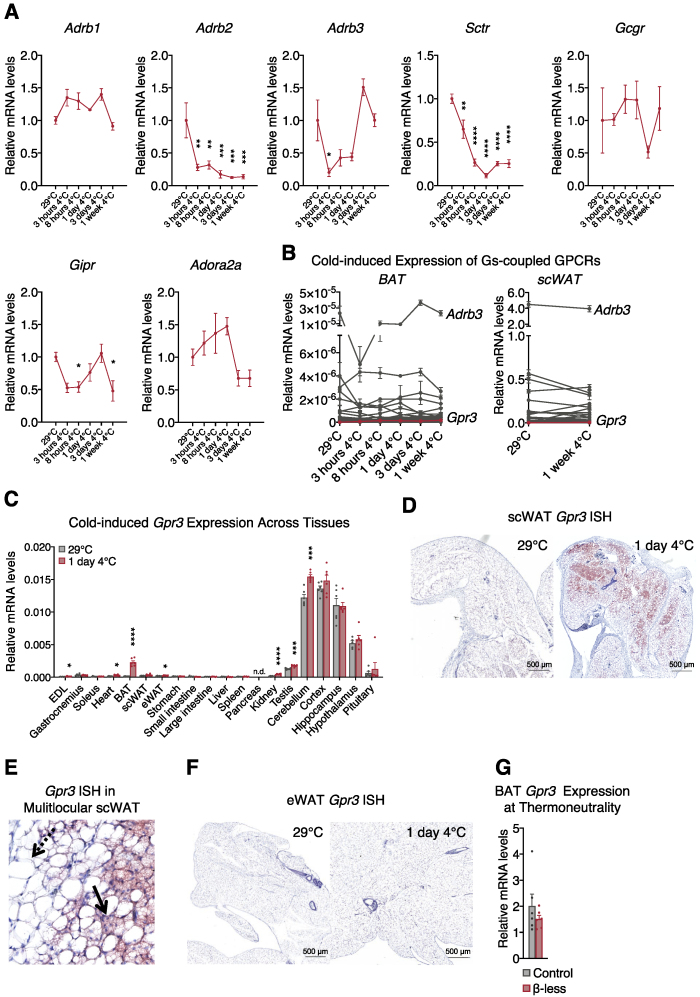
Cold-induced GPCR expression in mouse tissues and *Gpr3* transcription in β-less mice housed at thermoneutrality, related to Figures 1 and 2 (A) transcriptional regulation of established BAT activating Gs-coupled receptors in BAT during adaptation to cold. (B) induction of Gs-coupled receptors in brown (left) and subcutaneous (right) white adipose depots during adaptation to cold (non-normalized values from Figure 1B). (C) tissue panel of cold-induced *Gpr3* expression. (D) *in situ* hybridization (ISH) of *Gpr3* mRNA (red) in scWAT, E, scWAT (high magnification. Dotted arrow: Unilocular adipocyte. Solid arrow: Multilocular adipocyte), and, F, eWAT of thermoneutral-housed or cold-challenged mice. BAT *Gpr3* expression in, G, thermoneutral-acclimated β-less mice and wildtype controls. For all panels, error bars represent ±SEM, p ≤ 0.05=∗, p ≤ 0.01 = ∗∗, p ≤ 0.001 = ∗∗∗, p ≤ 0.0001 = ∗∗∗∗, t test (C) or Bonferroni's multiple comparisons test (A).

Given that GPCRs are under-represented in global pools of transcripts (Fredriksson and Schiöth, 2005), we employed a targeted qPCR array strategy to assess receptor expression over the course of cold adaptation in mice, focusing on the thermogenic-activating Gs-coupled family. Of the 44 Gs-coupled receptors examined, the one most profoundly cold-induced was *Gpr3* (Figures 1B, S1A and S1B; Table S1). *Gpr3* was also the most cold-induced Gs-coupled receptor in subcutaneous white adipose tissue (scWAT) (Figures 1B and S1B; Table S2), a depot that contains thermogenically competent beige adipocytes (Harms and Seale, 2013).

GPR3 is characterized by high intrinsic receptor activity that signals in the absence of an exogenous ligand (Eggerickx et al., 1995). Hence, overexpressing *Gpr3* at increasing concentrations is sufficient to stimulate cAMP production in a pattern similar to a ligand dose-response curve (Figure 1C). This constitutive activity is coupled exclusively to Gs proteins and cAMP production and is abolished in GPR3 constructs in which the DRY motif, responsible for G protein interaction, is mutated (Figures 1D and 1E). Yet, how GPR3 mediates ligand-independent constitutive Gs-coupling is unknown. Intrinsic activity of other GPCRs is heavily influenced by the N terminus (Brüser et al., 2016; Srinivasan et al., 2004; Toyooka et al., 2009). Accordingly, consecutive truncations of the GPR3 N terminus revealed that the region containing amino acids 18–27 was the most crucial for intrinsic signaling activity (Figures 1F and 1G). Interestingly, treating cells with a peptide fragment comprised only of amino acids 18–27 dose-dependently activated GPR3 (Figure 1H), but not the closely related cannabinoid 1 receptor (CB1) (Figure 1I). These data show that the GPR3 N terminus confers constitutive activity and, thus, transcriptional induction of *Gpr3* essentially serves as the *de facto* activation event compared to ligand-binding of conventional GPCRs.

We next investigated how cold exposure regulated *Gpr3* transcription across mouse tissues. Basal expression was highest in the CNS (Figure S1C), consistent with earlier reports (Eggerickx et al., 1995); however, *Gpr3* was most strikingly cold-induced in BAT compared to all other tissues surveyed (Figure 1J). These cold-mediated increases in *Gpr3* mRNA were specifically observed in the mature brown (Figures 1K and 1L) and beige adipocyte populations (Figures S1D–S1F). Thus, transcriptional induction of the constitutively active, Gs-coupled receptor, *Gpr3*, represents a hallmark of thermogenic adipocyte activation.

### A lipolytic signal controls cold-induced expression of *Gpr3*

**Figure 2:**
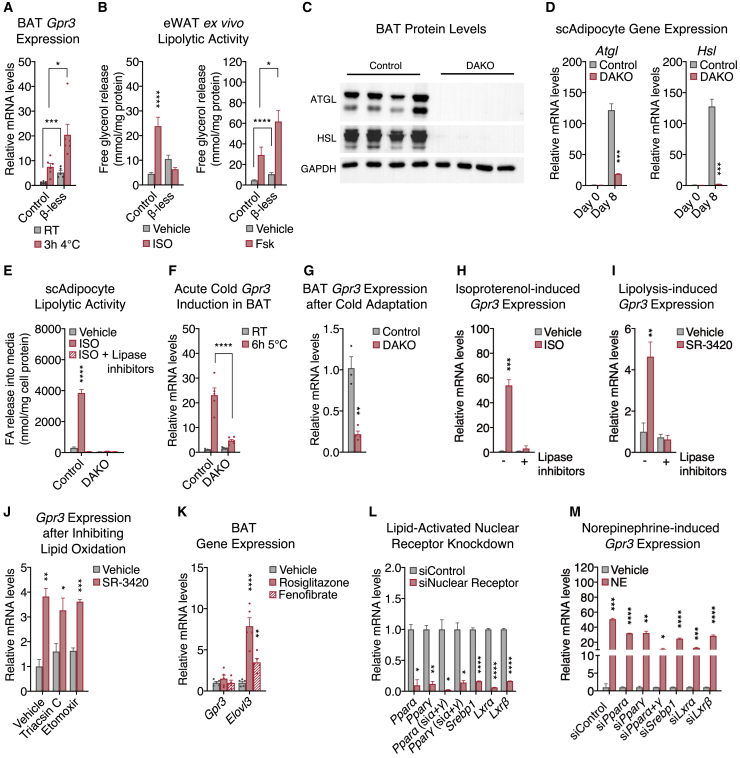
A lipolytic signal controls cold-induced expression of *Gpr3* (A) Cold-induced *Gpr3* expression in BAT. (B) Lipolytic activity in *ex vivo* eWAT from wild-type control and β-less mice stimulated with either isoproterenol (ISO) or forskolin (Fsk). (C–E) (C) Protein levels of adipose triglyceride lipase (ATGL) and hormone-sensitive lipase (HSL) in BAT, (D) gene expression of *Atgl* and *Hsl*, and (E) lipolytic activity in primary subcutaneous white adipocytes following ISO treatment with or without lipase inhibitors (ATGL inhibitor, Atglistatin; HSL inhibitor, 76-0079). (F and G) BAT *Gpr3* expression in (F) acute cold-induced (RT, room temperature) and (G) 3-week cold-adapted DAKO mice and control littermates. (H) *Gpr3* expression in primary brown adipocytes following ISO treatment with or without lipase inhibitors (ATGL inhibitor, Atglistatin; HSL inhibitor, CAY10499). (I) Regulation of brown adipocyte *Gpr3* expression by the lipolytic activator, SR-3420, with or without lipase inhibitors (ATGL inhibitor, Atglistatin; HSL inhibitor, CAY10499). (J) *Gpr3* expression in brown adipocytes following SR-3420 treatment with or without lipid oxidation inhibitors. (K) BAT gene expression from mice given PPARα (fenofibrate) or PPARγ (rosiglitazone) agonists for 2 weeks. (L) Small interfering RNA (siRNA)-mediated knockdown of the lipid-activated nuclear receptors. (M) Impact on norepinephrine (NE)-induced *Gpr3* expression in brown adipocytes. For all panels, error bars represent ±SEM, p ≤ 0.05 = ∗, p ≤ 0.01 = ∗∗, p ≤ 0.001 = ∗∗∗, p ≤ 0.0001 = ∗∗∗∗, t test (A, B, D, F–J, L, and M) or Bonferroni's multiple comparisons test (E and K). See also Figure S1.

Cold exposure classically regulates BAT gene expression through norepinephrine (NE)-mediated activation of β-adrenergic receptors (Cannon and Nedergaard, 2004; Collins, 2012). Yet, alternate mechanisms exist that activate cold-induced thermogenesis in the absence of β-adrenergic signaling (Chen et al., 2019; Razzoli et al., 2015). To determine the β-adrenergic dependence of cold-regulated *Gpr3* expression, we used mice in which ADRB1, ADRB2, and ADRB3 were genetically ablated (the so-called β-less mice) (Bachman et al., 2002; Razzoli et al., 2015). Cold-induced *Gpr3* transcription in BAT was not only preserved in the β-less mice but was significantly higher than controls (Figure 2A). Loss of β-adrenergic receptors even increased basal expression of BAT *Gpr3* at room temperature (Figure 2A). Housing at 30°C normalized *Gpr3* expression to wild-type levels (Figure S1G), indicating that the nonadrenergic control of *Gpr3* was dependent on cold stress. Therefore, we set out to identify the pathway responsible for cold-regulation of *Gpr3* expression. Given that adipose tissue lipolysis can be initiated through nonadrenergic means (Braun et al., 2018) and can modulate brown adipocyte gene expression (Mottillo et al., 2012), we assessed lipolytic activity in the β-less mice. As expected, white adipocytes from wild-type but not β-less mice responded to isoproterenol (ISO) (Figure 2B). However, basal and forskolin-stimulated lipolysis was higher in β-less eWAT compared to wild-types (Figure 2B). These findings mirrored the patterns of basal and cold-induced *Gpr3* expression in BAT from wild-type and β-less mice, leading us to speculate that lipolytic signals might influence *Gpr3* transcription.

In order to assess a causal role of lipolysis in *Gpr3* induction, mice were generated in which the two key lipolytic enzymes, adipose triglyceride lipase (ATGL) and hormone sensitive lipase (HSL) (Zechner et al., 2012), were conditionally ablated in adipocytes using AdipoQ-Cre (hereby referred to as DAKO) (Figures 2C and 2D), effectively eliminating lipolytic activity (Figure 2E). Acute cold induction of *Gpr3* expression in BAT was significantly blunted in lipolysis-deficient DAKO mice compared to control littermates (Figure 2F). This attenuated transcription was still observed after 3 weeks of cold adaptation (Figure 2G). Collectively, these findings suggest a critical role for adipose lipolysis in the physiological regulation of BAT *Gpr3*. In primary brown adipocytes, adrenergic agonism by ISO recapitulated the cold induction of *Gpr3* expression (Figure 2H). Yet, this effect was largely blocked by ATGL and HSL inhibitors (Figure 2H), suggesting that adrenergic regulation of *Gpr3* was independent of the canonical protein kinase A (PKA)-CREB cascade. Moreover, bypassing adrenergic stimulation altogether and directly triggering lipolysis by the pharmacological compound SR-3420, which blocks ABHD5 and PLIN1 interaction (Rondini et al., 2017; Sanders et al., 2015), was sufficient to increase *Gpr3* transcription (Figure 2I). Inhibition of fatty acid activation (i.e. acyl-CoA synthetase) or mitochondrial transport (i.e. carnitine palmitoyl-transferase) did not affect lipolysis-induced *Gpr3* expression (Figure 2J), indicating that lipolysis-derived signals did not require further metabolic processing to exert transcriptional control. These data reveal that a lipolytic signal acts as a direct, cell autonomous driver of *Gpr3* expression in brown adipocytes.

Lipolytic products have previously been shown to increase expression of brown adipocyte genes linked to fatty acid oxidation through the PPAR family of transcriptional activators (Mottillo et al., 2012). Yet neither PPARα nor PPARγ agonists affected *Gpr3* expression in BAT (Figure 2K). Moreover, NE induction of *Gpr3* in brown adipocytes was fully preserved after knockdown of PPARα and PPARγ, as well as several other known lipid-activated nuclear receptors (Figures 2L and 2M). Thus, we conclude that a lipolytic signal mediates *Gpr3* transcriptional control in a manner distinct from classic lipid and cAMP-PKA-CREB regulation.

### *Gpr3* overexpression activates the adipose thermogenic program independently of sympathetic signaling

**Figure S2:**
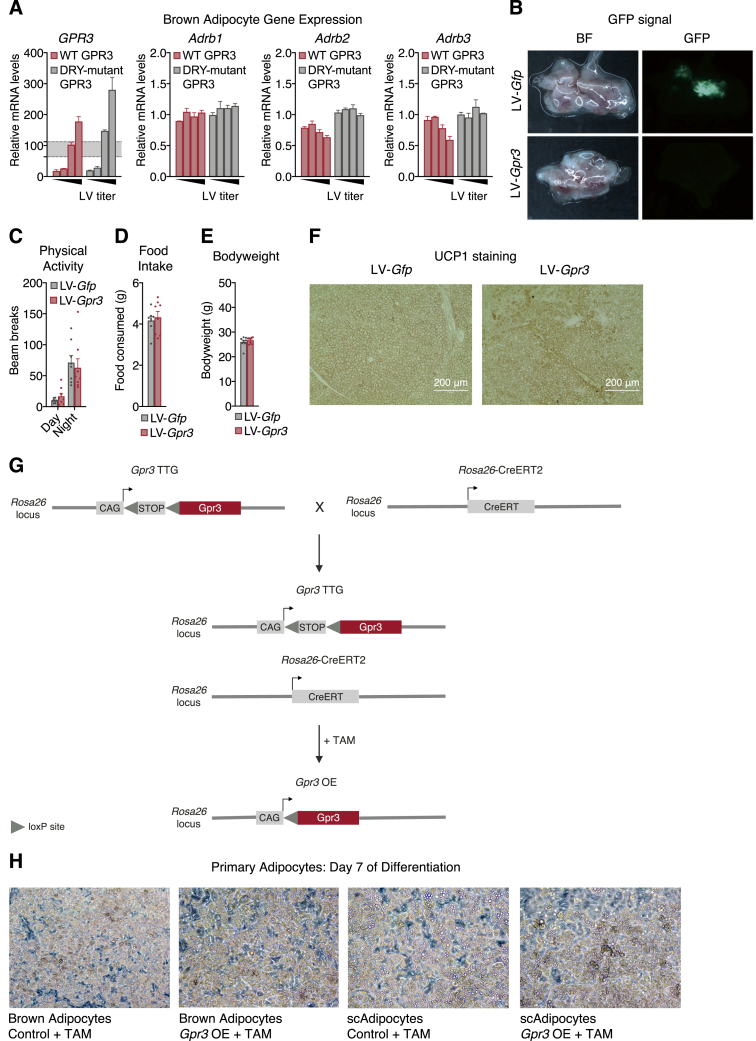
Characterization of the *in vitro* and *in vivo* LV delivery models and *Gpr3* OE primary adipocyte model, related to Figure 3 (A) gene expression from brown adipocytes following lentiviral (LV)-mediated overexpression of wildtype (WT) and DRY-mutant GPR3. The shaded region indicates the physiological range of maximal cold-induced *Gpr3* expression in BAT. (B–F) (B) Fluorescent visualization of BAT (BF=bright field), (C) physical activity, (D) food intake, (E) bodyweight, and (F) UCP1 staining in BAT from mice injected with LV particles carrying either *Gfp* or *Gpr3*. (G) schematic of *Gpr3* OE mice, in which the *Gpr3* coding region is preceded by a synthetic CAG promoter and lox-STOP-lox cassette (TAM=tamoxifen). (H) representative light microscopy images of primary brown and subcutaneous white adipocytes with and without TAM-induced *Gpr3* expression. For all panels, error bars represent ±SEM.

**Figure 3:**
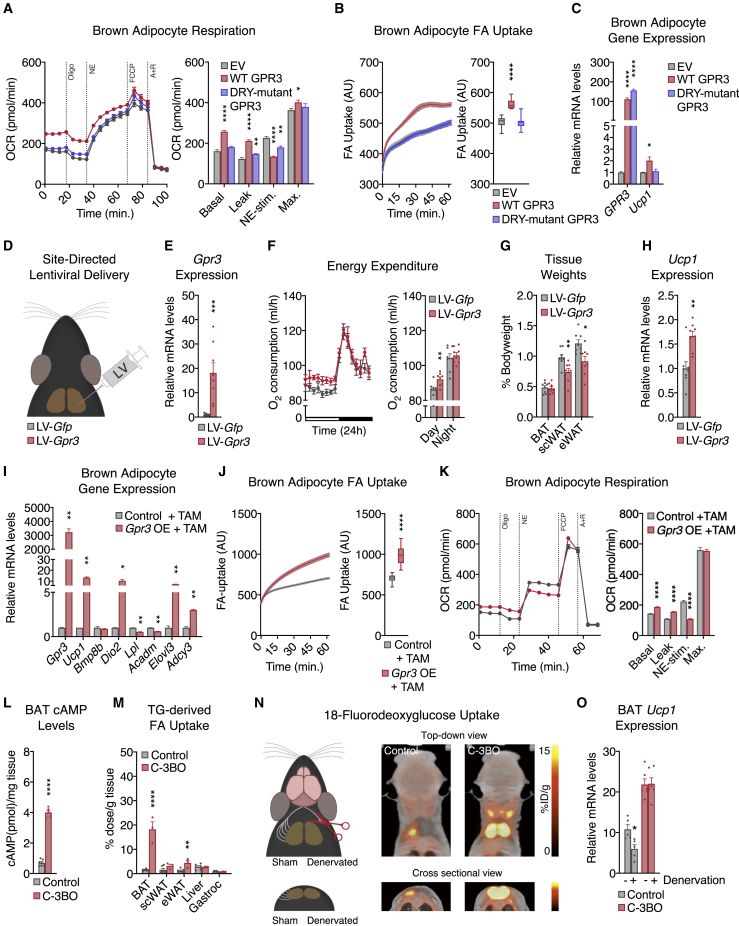
*Gpr3* overexpression activates the adipose thermogenic program independently of sympathetic signaling (A–C) (A) Mitochondrial respiration, (B) fatty acid (FA) uptake, and (C) gene expression from brown adipocytes expressing wild-type (WT) or DRY-mutant GPR3. (D–H) (D) Schematic depicting the site-directed, high-titer lentiviral (LV) injections used to (E) overexpress *Gfp* or *Gpr3* in BAT and assess (F) energy expenditure, (G) tissue weights, and (H) *Ucp1* gene expression. (I–K) (I) Gene expression, (J) FA uptake, and (K) mitochondrial respiration of primary brown adipocytes with or without tamoxifen (TAM)-induced *Gpr3* expression. (L and M) (L) BAT cAMP levels and (M) tissue-specific triglyceride (TG)-derived FA uptake in C-3BO mice and control littermates. (N and O) (N) Schematic of BAT denervation protocol used to assess 18-fluorodeoxyglucose (^18^F-FDG) uptake and (O) *Ucp1* gene expression of C-3BO mice and control littermates. For all panels, error bars represent ±SEM, p ≤ 0.05 = ∗, p ≤ 0.01 = ∗∗, p ≤ 0.001 = ∗∗∗, p ≤ 0.0001 = ∗∗∗∗, t test (E–M and O) or Bonferroni's multiple comparisons test (A–C). Box plots are presented as box: 25^th^ to 75^th^ percentile, and whiskers: min to max. See also Figures S2 and S3.

To evaluate the functional consequences of *Gpr3* cold induction, wild-type *Gpr3* and the Gs-signaling-deficient DRY mutant were overexpressed using lentiviral vectors in mature brown adipocytes at levels comparable to those elicited by physiological cold (Figure S2A). Wild-type, but not mutant GPR3, increased cellular energy expenditure (Figure 3A), fatty acid uptake (Figure 3B), and *Ucp1* gene expression (Figure 3C). Surprisingly, wild-type GPR3 also decreased NE-induced respiration (Figure 3A) and β-adrenergic receptor gene expression (Figure S2A), suggesting a potential counter-regulation between constitutive and inducible Gs-coupled receptors. We next investigated how these cell-autonomous actions of GPR3 impacted BAT function *in vivo*. Lentiviral particles carrying *Gpr3* (LV-*Gpr3*) were directly injected into BAT (Figures 3D and S2B), resulting in 16.2-fold overexpression of *Gpr3* compared to mice injected with *Gfp* control virus (LV-*Gfp*) (Figure 3E). LV-*Gpr3*-injected mice exhibited significantly higher oxygen consumption (Figure 3F) and decreased WAT weights (Figure 3G) without differences in motility, food intake, or overall bodyweight (Figures S2C–S2E). Moreover, *Ucp1* gene expression and protein levels were elevated in the BAT from LV-*Gpr3* mice compared to BAT of LV-*Gfp* mice (Figures 3H and S2F). These findings show that transcriptional induction of *Gpr3* is fully sufficient to orchestrate the hallmarks of thermogenesis in cells and mice.

**Figure S3:**
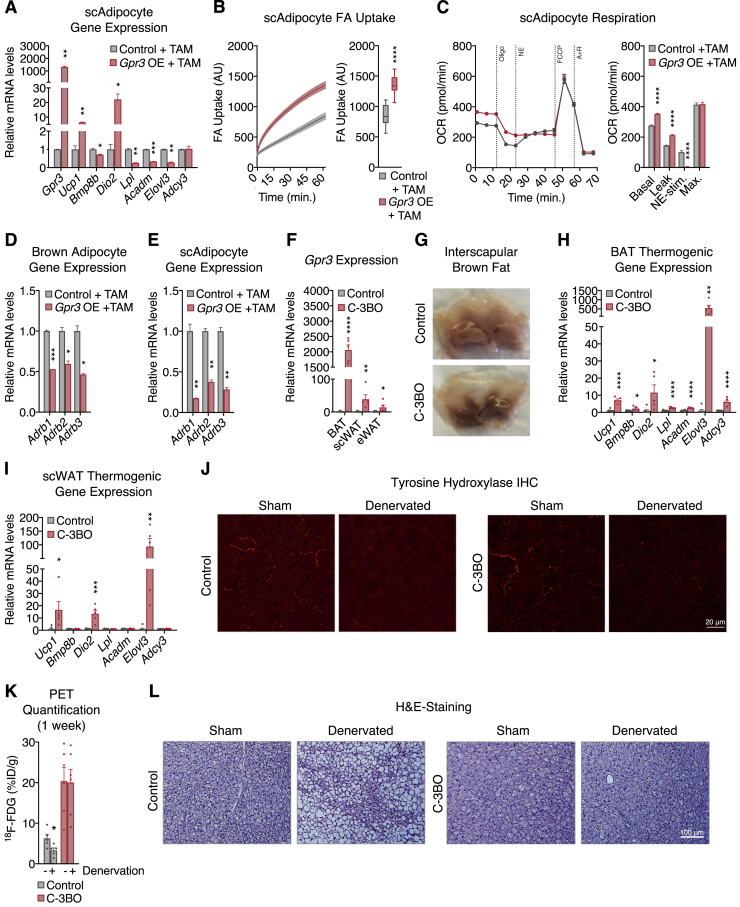
Characterization of the *Gpr3* OE primary adipocyte model (continued) and C-3BO mouse model, related to Figure 3 (A–C) (A) thermogenic gene expression, (B) fatty acid (FA) uptake, and (C) mitochondrial respiration of primary subcutaneous white adipocytes with and without TAM-induced *Gpr3* expression. (D and E) Adrenergic receptor gene expression from primary (D) brown and (E) subcutaneous white adipocytes with and without TAM-induced *Gpr3* expression. (F–I) (F) *Gpr3* expression levels across fat depots, (G) representative interscapular BAT (iBAT) images, (H) thermogenic gene expression in BAT, and (I) thermogenic gene expression in scWAT of chow-fed C-3BO mice and control littermates. (J–L) (J) tyrosine hydroxylase immunohistochemistry (IHC), (K) quantified 18-Fluorodeoxyglucose (^18^F-FDG) uptake in BAT one week after denervation surgery (PET=positron emission tomography), and (L) hematoxylin and eosin (H&E)-staining of sham and denervated iBAT of C-3BO mice and control littermates. For all panels, error bars represent ±SEM, p ≤ 0.05 = ∗, p ≤ 0.01 = ∗∗, p ≤ 0.001 = ∗∗∗, p ≤ 0.0001 = ∗∗∗∗, t test. Box plots are presented as box: 25^th^ to 75^th^ percentile and whiskers: min to max.

We then developed a conditional gain-of-function model (hereby referred to as *Gpr3* TTG) (Figure S2G) for robust and sustained genetic manipulation of *Gpr3 in vitro* and *in vivo. Gpr3* TTG mice were crossed with *Rosa26*-CreERT2 animals to facilitate tamoxifen-inducible overexpression of *Gpr3* (hereby referred to as *Gpr3* OE) (Figure S2G) in isolated primary brown and subcutaneous white adipocytes (Figure S2H). Tamoxifen-triggered *Gpr3* overexpression significantly increased the expression of thermogenic genes (Figures 3I and S3A), fatty acid uptake (Figures 3J and S3B), and basal and leak mitochondrial respiration (Figures 3K and S3C). Similar to our lentiviral cell studies, *Gpr3* overexpression in this primary adipocyte model blunted NE-induced respiration (Figures 3K and S3C) and suppressed expression of the β-adrenergic receptors (Figures S3D and S3E), further supporting a counter-regulatory interaction between GPR3 and other Gs-coupled receptors.

We next crossed *Gpr3* TTG mice with *Ucp1*-Cre animals (Kong et al., 2014) to generate constitutive *Gpr3* brown and beige adipocyte overexpressors (hereby referred to as C-3BO) (Figure S3F). Consistent with its function as a constitutively active Gs-coupled receptor, increasing *Gpr3* expression led to a 5- to 6-fold elevation in basal cAMP levels in C-3BO BAT (Figure 3L). At thermoneutrality, where the sympathetic tone in adipose tissue is lowest, BAT from C-3BO mice displayed a distinctly darker brown color than controls, implying increased mitochondrial density and/or less TG content (Figure S3G). Accordingly, thermogenic genes, such as *Ucp1*, were elevated in both brown and subcutaneous fat depots (Figures S3H and S3I). Given the physiological role of BAT in TG clearance (Bartelt et al., 2011; Hoeke et al., 2016), we determined the direct influence of GPR3 on adipose lipid uptake. BAT from C-3BO mice took up significantly more TG-derived fatty acids compared to control littermates and all other tissues measured (Figure 3M). BAT metabolic activity was further evaluated using ^18^F-fluorodeoxyglucose (^18^F-FDG) uptake by positron emission tomography (PET) coupled with computed tomography (CT). We carried out unilateral denervation of the interscapular BAT depot in which the right lobe was surgically denervated while the left lobe remained intact (Figures 3N and S3J). In the intact BAT lobes, ^18^F-FDG uptake was significantly higher in C-3BO mice compared to littermate controls (Figures 3N and S3K). Strikingly, GPR3 overexpression was sufficient to sustain elevated BAT glucose uptake (Figures 3N and S3K) and *Ucp1* gene expression (Figure 3O) as well as resist lipid accumulation (Figure S3L) in the sympathetically denervated BAT lobes. Thus, GPR3 drives BAT metabolic activity independently of canonical sympathetic nervous system signaling.

### Dietary fat potentiates GPR3-mediated thermogenic activation

**Figure S4:**
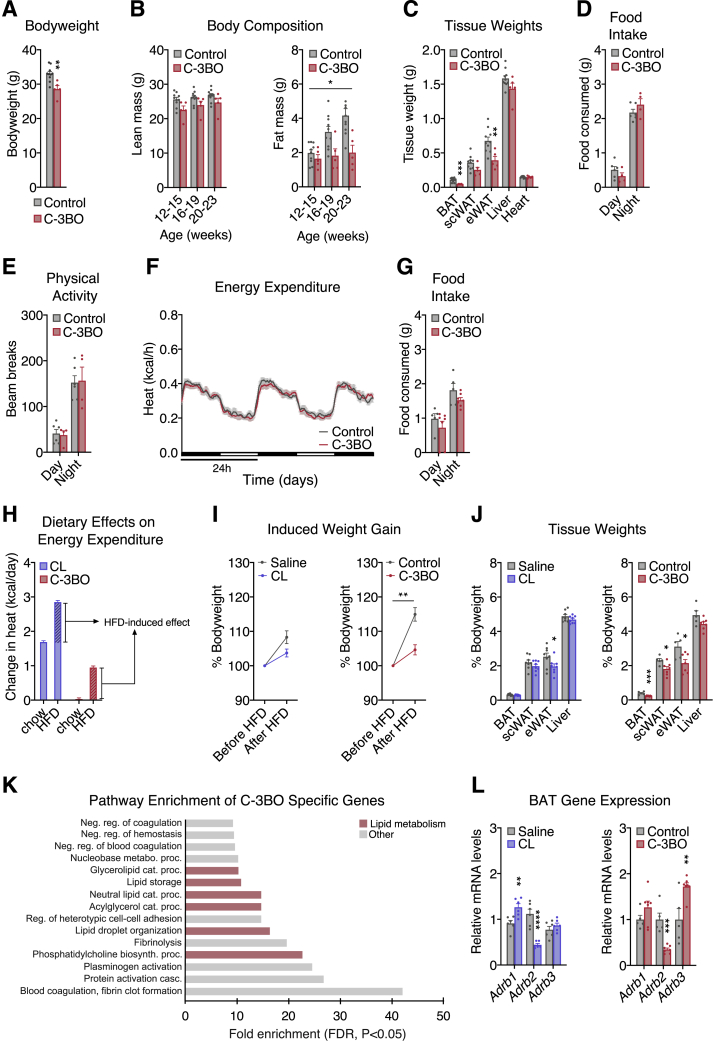
Phenotyping of the C-3BO mouse model, related to Figure 4 (A–F) (A) bodyweights, (B) lean and fat mass, (C) tissue weights, (D) food intake (average per day), (E) physical activity (average per 15 min), and (F) energy expenditure of chow-fed C-3BO mice and control littermates. (G) food intake (average per day) of C-3BO mice and control littermates after transition to high fat diet (HFD). (H) change in HFD-induced energy expenditure between CL-316,243 (CL) and saline-injected mice and C-3BO and control littermates. (I and J) (I) bodyweight gain and (J) tissue weights of CL/saline and C-3BO/control cohorts (after 1-week HFD-challenge). (K) pathway analysis of gene networks specifically induced in C-3BO mice. (L) gene expression of CL/saline and C-3BO/control cohorts (after 1-week HFD-challenge). For all panels, error bars represent ±SEM, p ≤ 0.05 = ∗, p ≤ 0.01 = ∗∗, p ≤ 0.001 = ∗∗∗, t test (A, C, J, and L), two-way ANOVA (B and I), or Fisher’s exact test (K).

**Figure 4:**
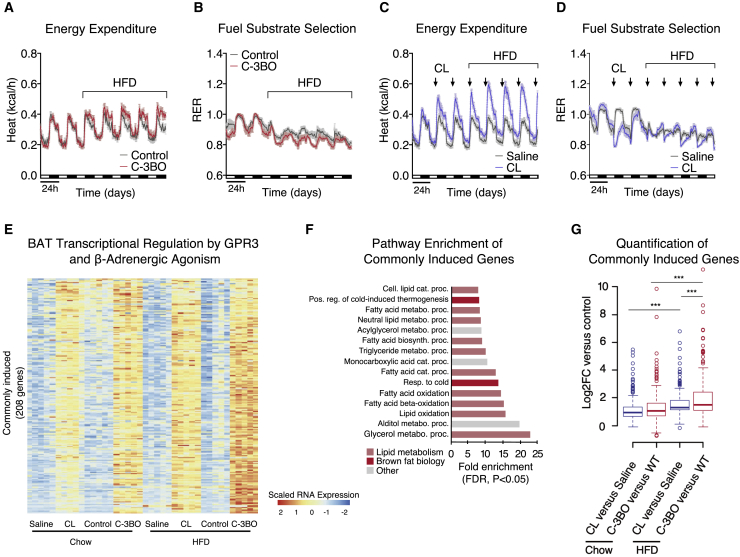
Dietary fat potentiates GPR3-mediated thermogenic activation (A–D) Indirect calorimetry and respiratory exchange ratio (RER) during the transition from chow to high fat diet (HFD) for (A and B) C-3BO mice and control littermates and (C and D) mice injected daily with 1 mg/kg CL-316,243 (CL). (E–G) (E) Heat map, (F), pathway enrichment, and (G) quantification of genes induced by CL-treatment and in C-3BO mice under chow and HFD-fed conditions. Error bars represent ±SEM, p ≤ 0.001 = ∗∗∗, Fisher’s exact test (F) or Wilcoxon signed-rank tests (G). See also Figure S4.

Consistent with the GPR3-mediated increase in basal BAT activation, C-3BO mice had lower body and tissue weights than control littermates on chow diet (Figures S4A–S4C). Yet, there were no observable changes in food intake, physical activity, or whole-body energy expenditure (Figures S4D–S4F). In striking contrast to chow, transitioning to high fat diet (HFD) robustly increased energy expenditure in C-3BO mice within the first day and throughout the remainder of the dietary challenge (Figure 4A). Despite elevated calorie-burning, C-3BOs consumed the same total amount of food as littermate controls (Figure S4G). However, the respiratory exchange ratio (RER) of C-3BO mice dropped even lower than that of controls following the transition to HFD (Figure 4B), suggesting that GPR3-activated oxygen consumption was preferentially fueled by lipids. These findings reveal that dietary lipids amplify GPR3-dependent adipose thermogenesis *in vivo* to modulate systemic metabolism.

We next compared GPR3-mediated BAT activation with the canonical mode of regulation via β-adrenergic signaling. In order to specifically target β-adrenergic signaling in thermogenic adipocytes, we used the selective β3-adrenergic agonist, CL-316,243 (hereby referred to as CL). Unlike increased *Gpr3* expression, we found that daily CL injections markedly stimulated energy expenditure on chow diet (Figure 4C). Interestingly, however, HFD boosted CL-induced calorie-burning to a similar magnitude as it did in C-3BO mice (Figure S4H). Yet, CL treatment only moderately lowered RER, indicating that GPR3 thermogenic activation more specifically favored lipid oxidation (Figure 4D). Despite these different calorimetric profiles, β-adrenergic agonism and GPR3 gain-of-function both prevented bodyweight gain and adipose expansion to a similar degree (Figures S4I–S4J). Thus, constitutive GPR3-mediated BAT activation improves metabolic homeostasis comparably to induced sympathetic signaling but with a preference for lipid as a fuel.

Sustained activation of adipose thermogenesis is supported by global remodeling of gene networks (Marcher et al., 2015). We sought to compare the transcriptional changes from C-3BO and CL-treated mice on chow and HFD. In total, 270 genes were induced by repeated CL injections in either chow or HFD-fed animals, of which 208 were shared by GPR3 overexpression under the same conditions (log2FC >1, pAdjusted <0.05) (Figure 4E). These commonly induced genes were enriched for biological processes related to lipid metabolism and cold-induced thermogenesis (Figure 4F). There were an additional 307 genes significantly induced by GPR3, but not CL, which were also enriched for lipid-related pathways (Figure S4K). However, it is unclear whether these GPR3 selective genes are due to *bona fide* receptor-specific signaling or to potential differences in cAMP accumulation between GPR3 and CL-induced activity. Consistent with our cellular gain-of-function findings on transcriptional interaction between constitutive and inducible Gs-coupled receptors, *Gpr3* overexpression in BAT significantly altered β-adrenergic receptor expression (Figure S4L). Notably, commonly induced genes were increased to a greater magnitude on HFD and significantly boosted in C-3BO mice compared to CL-treated mice in this condition (Figure 4G). Thus, dietary fat enhances GPR3-mediated upregulation of gene networks linked to lipid utilization and adipose thermogenesis.

### GPR3 activation of thermogenic adipocytes counteracts metabolic disease

**Figure 5:**
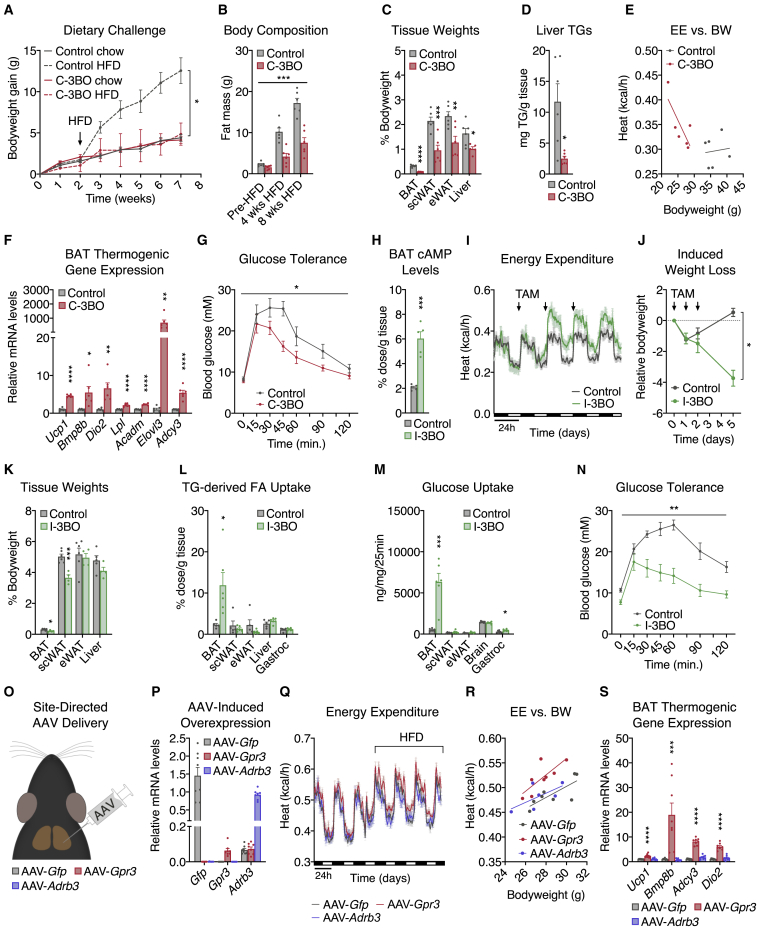
GPR3 activation of thermogenic adipocytes counteracts metabolic disease (A and B) (A) Bodyweight (BW) gain and (B) body composition of C-3BO mice and control littermates over the course of an 8-week high fat diet (HFD) challenge. (C–G) (C) Tissue weights, (D) liver triglycerides (TG), (E) energy expenditure (EE), (F) BAT thermogenic gene expression, and (G) glucose tolerance of C-3BO mice and control littermates during HFD challenge. (H and I) (H) cAMP levels in BAT 1 week after tamoxifen (TAM) administration, and (I) indirect calorimetry of obese I-3BO mice and control littermates following 3 consecutive days of TAM-treatment by oral gavage. (J and K) (J) Weight loss and (K) tissue weights 1 week after TAM-administration. (L–N) (L) TG-derived fatty acid (FA) uptake, (M) glucose uptake, and (N) glucose tolerance in HFD-fed I-3BO mice and control littermates. (O–R) (O) Schematic depicting the site-directed adeno-associated virus (AAV) injections used to (P) overexpress *Gfp*, *Gpr3,* or *Adrb3* in BAT and assess, (Q) energy expenditure during chow to HFD transition, (R) HFD-induced EE versus BW, and (S) BAT thermogenic gene expression. For all panels, error bars represent ±SEM, p ≤ 0.05 = ∗, p ≤ 0.01 = ∗∗, p ≤ 0.001 = ∗∗∗, p ≤ 0.0001 = ∗∗∗∗, t test (C, D, F, H, K, L, and M), Bonferroni's multiple comparisons test (S), or two-way ANOVA (A, B, G, J, and N). See also Figure S5.

**Figure S5:**
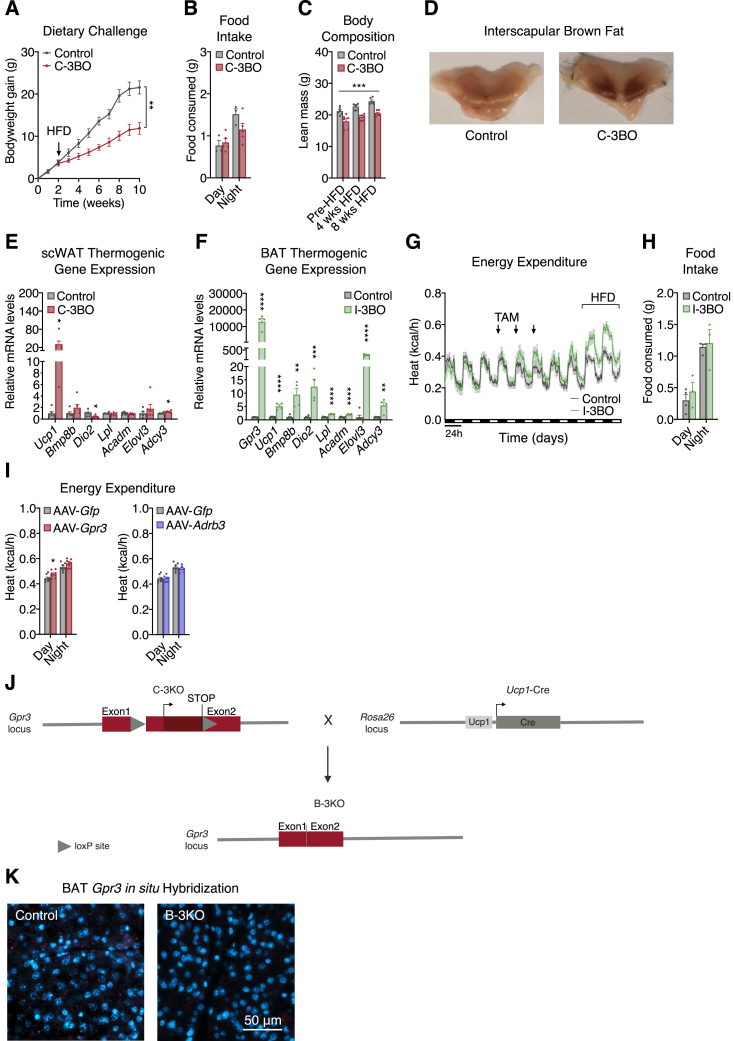
Characterization of the C-3BO, I-3BO, AAV-modified, and B-3KO mouse models, related to Figures 5 and 6 (A) bodyweight gain of C-3BO mice and control littermates challenged with high fat diet (HFD) (independent experiment from the study in Figure 3). (B–E) (B) food intake (average per day), (C) lean mass, (D) representative interscapular BAT images, and (E) thermogenic gene expression in scWAT of HFD-fed C-3BO mice and control littermates. (F) thermogenic gene expression in BAT of HFD-fed I-3BO mice and control littermates. (G and H) (G) HFD-induced energy expenditure and (H) food intake (average per day) of HFD-fed I-3BO mice and control littermates. (I) HFD-induced day and night energy expenditure in mice infected with adeno-associated virus (AAV) particles carrying either *Gfp*, *Adrb3*, or *Gpr3*. (J) schematic for conditional deletion of *Gpr3* in the B-3KO mouse model. (K) *Gpr3 in situ* hybridization in BAT from B-3KO and control littermates. For all panels, error bars represent ±SEM, p ≤ 0.05 = ∗, p ≤ 0.01 = ∗∗, p ≤ 0.001 = ∗∗∗, p ≤ 0.0001 = ∗∗∗∗, t test (E, F, and I) or two-way ANOVA (A and C).

Chronic pharmacological β-adrenergic stimulation maintains elevated BAT thermogenesis to improve systemic energy homeostasis and counteract metabolic disease (Berbée et al., 2015; Xiao et al., 2015). Therefore, we next investigated the physiological impact of GPR3-dependent BAT activation under chronic obesogenic conditions. We found that C-3BO mice were completely protected from developing diet-induced obesity (Figures 5A and S5A) despite maintaining comparable levels of food intake (Figure S5B). The dramatic bodyweight difference between C-3BO mice and control littermates was due to reduced adipose expansion and accompanied by decreased ectopic fat deposition in the liver (Figures 5B–5D and S5C). C-3BO mice maintained elevated whole-body energy expenditure (Figure 5E) as well as darker brown BAT depots (Figure S5D) and higher thermogenic gene expression (Figures 5F and S5E) throughout the HFD challenge. Consistent with lower adiposity and increased calorie-burning, C-3BO mice were also more glucose tolerant than control animals (Figure 5G). Thus, GPR3 overexpression in thermogenic adipocytes completely protects mice from the development of metabolic disease.

The C-3BO model results in *Gpr3* overexpression in brown and beige adipocytes beginning early in life of the animal and, therefore, is less reflective of the acute transcriptional regulation by cold. To more accurately mimic *Gpr3* cold induction, we crossed *Gpr3* TTG mice with *Ucp1*-CreERT2 animals (Rosenwald et al., 2013) to generate tamoxifen-inducible *Gpr3* brown and beige adipocyte overexpressors (hereby referred to as I-3BO). Acute induction of *Gpr3* produced a thermogenic gene profile (Figure S5F) and HFD-catalyzed energy expenditure (Figure S5G) similar to C-3BO mice (Figures 4A and 5F). To test the ability of GPR3 to reverse metabolic dysfunction, we next rendered I-3BO mice and control littermates obese on HFD before tamoxifen administration. *Gpr3* induction in thermogenic adipocytes of obese mice increased BAT cAMP levels 2- to 3-fold and robustly boosted whole-body energy expenditure without affecting food intake (Figures 5H, 5I, and S5H). After 1 week of GPR3 activation, I-3BO mice had significantly lower bodyweights and scWAT mass (Figures 5J and 5K) as well as elevated TG-derived fatty acid and glucose uptake into BAT compared to controls (Figures 5L and 5M). These targeted increases in BAT function dramatically improved whole-body glycemic control (Figure 5N). Therefore, mimicking acute cold induction of *Gpr3* in thermogenic adipocytes counteracts metabolic dysfunction and restores systemic energy homeostasis in mice.

Our genetic gain-of-function studies suggested that GPR3 may hold therapeutic potential for metabolic disease. In particular, the intrinsic, constitutive activity of this receptor makes it an appealing gene therapy candidate as GPR3 can sustain adipose energy expenditure without continued administration of an exogenous ligand. To explore this concept, we designed three adeno-associated viruses (AAV) expressing *Gpr3*, *Gfp*, or *Adrb3*, each under the control of the proximal *Ucp1* promoter, and injected them directly into BAT depots of mice (Figures 5O and 5P). Five weeks after viral delivery, none of the AAVs affected energy expenditure on chow diet; however, in line with our genetic models, GPR3 significantly augmented HFD-induced calorie-burning (Figures 5Q, 5R, and S5I). Similarly, AAV-*Gpr3*, but not AAV-*Adrb3*, increased thermogenic gene programs in BAT (Figure 5S). Thus, in contrast to conventional GPCRs that require continued administration of ligands for pharmacological activation (Kobilka, 2007; Wettschureck and Offermanns, 2005), a single viral delivery of *Gpr3* to thermogenic adipose tissue provided sustained energy-expending capacity.

### BAT *Gpr3* is required for thermogenic activity *in vitro* but is compensated *in vivo*

**Figure 6:**
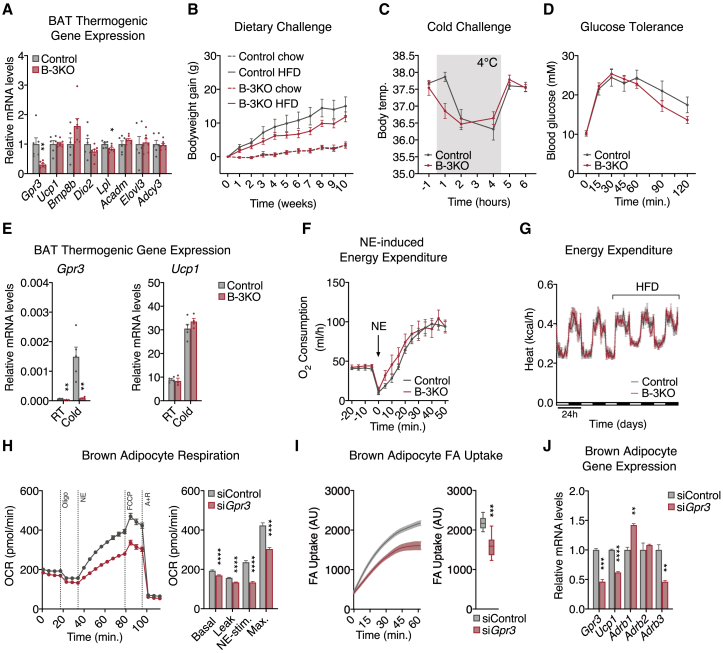
BAT *Gpr3* is required for thermogenic activity *in vitro* but is compensated *in vivo* (A) BAT thermogenic gene expression from high fat diet (HFD)-fed B-3KO mice and control littermates. (B–E) (B) HFD-induced weight gain, (C) cold tolerance, (D) glucose tolerance, and (E) cold-induced BAT gene expression from B-3KO mice and control littermates (RT, room temperature). (F) Norepinephrine (NE)-induced energy expenditure in anesthetized B-3KO mice and control littermates at thermoneutrality following acute cold-challenge. (G) Indirect calorimetry of B-3KO mice and control littermates during the transition from chow to HFD. (H–J) (H) Mitochondrial respiration, (I) fatty acid (FA) uptake, and (J) gene expression in brown adipocytes following siRNA-mediated *Gpr3* knockdown. For all panels, error bars represent ±SEM, p ≤ 0.01 = ∗∗, p ≤ 0.001 = ∗∗∗, p ≤ 0.0001 = ∗∗∗∗, t test. Box plots are presented as box: 25^th^ to 75^th^ percentile, and whiskers: min to max. See also Figure S5.

Our genetic and viral-mediated gain-of-function studies demonstrated that GPR3 was fully capable of driving the thermogenic program. However, whether BAT GPR3 is required for the activation of adipose thermogenesis remained unclear. A previous study found that whole-body *Gpr3* ablation reduced BAT thermogenesis and related gene programs in aged mice (Godlewski et al., 2015). To address specific contributions from *Gpr3* in thermogenic adipocytes, we generated BAT-specific *Gpr3* knockout mice (hereby referred to as B-3KO) (Figures S5J and S5K). There were no genotypic differences in thermogenic gene expression or propensity to develop HFD-induced obesity (Figures 6A–6D), suggesting that the global KO phenotypes originated from GPR3 in another tissue than BAT. Moreover, BAT thermogenic gene expression was unchanged between B-3KO mice and control littermates at room temperature or following acute cold exposure (Figure 6E). Neither NE nor HFD-induced energy expenditure was significantly affected by *Gpr3* deletion in BAT (Figures 6F and 6G). Collectively, these results are not surprising given that adipose thermogenesis can be activated even in mice globally lacking β-adrenergic receptors through compensatory measures (Chen et al., 2019; Razzoli et al., 2015). Thus, although our gain-of-function studies demonstrate that BAT GPR3 is sufficient, our loss-of-function studies show that it is not required for activation of adipose thermogenesis in mice.

Given that sympathetic tone and circulating factors can mask alternative thermogenic programs *in vivo* (Chen et al., 2019), we evaluated the cell autonomous role of *Gpr3* in brown adipocytes *in vitro*. Acute *Gpr3* depletion significantly decreased NE-induced oxygen consumption, fatty acid uptake, and *Ucp1* gene expression (Figures 6H–6J). In line with our earlier findings on *Gpr3* and β-adrenergic counter-regulation (Figures 2A, S3D, and S3E), transient knockdown of *Gpr3* robustly altered the expression of β-adrenergic receptors (Figure 6J), further supporting a coordinated interaction between the constitutive and inducible cAMP signaling programs. Taken together, these findings reveal that GPR3 regulates the thermogenic capacity of mouse brown adipocytes but this role appears to be compensated *in vivo*.

### GPR3 is an essential activator of human thermogenic adipocytes

**Figure 7:**
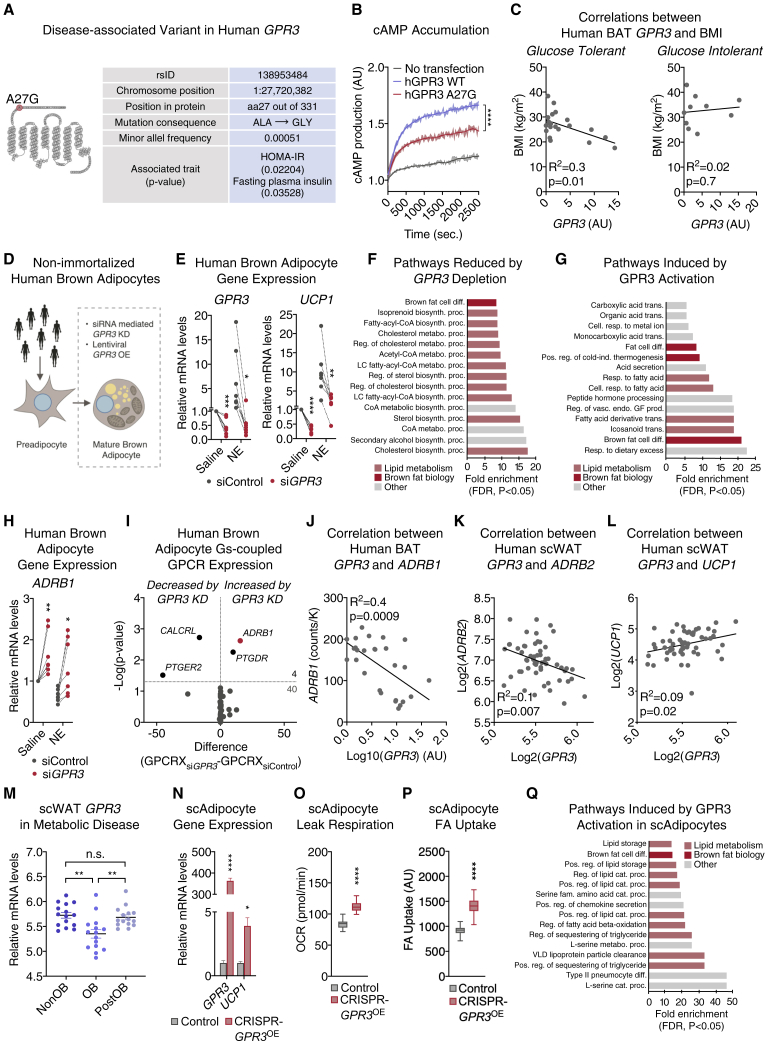
GPR3 is an essential activator of human thermogenic adipocytes (A) Structural location (snake plot) and disease association (table) of human *GPR3* variant, A27G. (B) Functional consequence of A27G mutation on GPR3 cAMP-inducing activity. (C) Correlation between BAT *GPR3* expression and body mass index (BMI) in glucose tolerant and glucose intolerant individuals. (D) Schematic of *GPR3* loss-of-function and gain-of-function studies in patient-derived, non-immortalized brown adipocytes. (E) Gene expression of siRNA-mediated *GPR3* knockdown and 4-h vehicle or norepinephrine (NE) treatment of patient-derived, non-immortalized brown adipocytes. (F and G) Pathway analysis of gene networks (F) reduced by *GPR3* depletion and (G) induced by GPR3 activation in patient-derived, non-immortalized brown adipocytes. (H) Gene expression following siRNA-mediated *GPR3* knockdown and 4-h vehicle or NE treatment of patient-derived, non-immortalized brown adipocytes. (I) Change in the gene expression of Gs-coupled GPCRs in human brown adipocytes following siRNA-mediated *GPR3* knockdown. (J) Correlation between *GPR3* and *ADRB1* expression in human BAT. (K and L) Correlation between (K) *GPR3* and *ADRB2* and (L) *GPR3* and *UCP1* expression in human scWAT. (M) *GPR3* expression in scWAT before and after bariatric surgery (NonOB, non-obese; OB, obese; PostOB, post-obese). (N–P) (N) Gene expression, (O) leak respiration, and (P) fatty acid (FA) uptake in human subcutaneous white adipocytes in which *GPR3* expression has been induced by CRISPR/Cas9-engineering. (Q) Pathway analysis of gene networks induced by GPR3 in CRISPR/Cas9-engineered human subcutaneous white adipocytes. For all panels, error bars represent ±SEM, p ≤ 0.05 = ∗, p ≤ 0.01 = ∗∗, p ≤ 0.001 = ∗∗∗, p ≤ 0.0001 = ∗∗∗∗, t test (E, H, I, and M–P), two-way ANOVA (B), simple linear regression (C and J–L), or Fisher’s exact test (F, G, and Q). Box plots are presented as box: 25^th^ to 75^th^ percentile, and whiskers: min to max. See also Figures S6 and S7.

**Figure S6:**
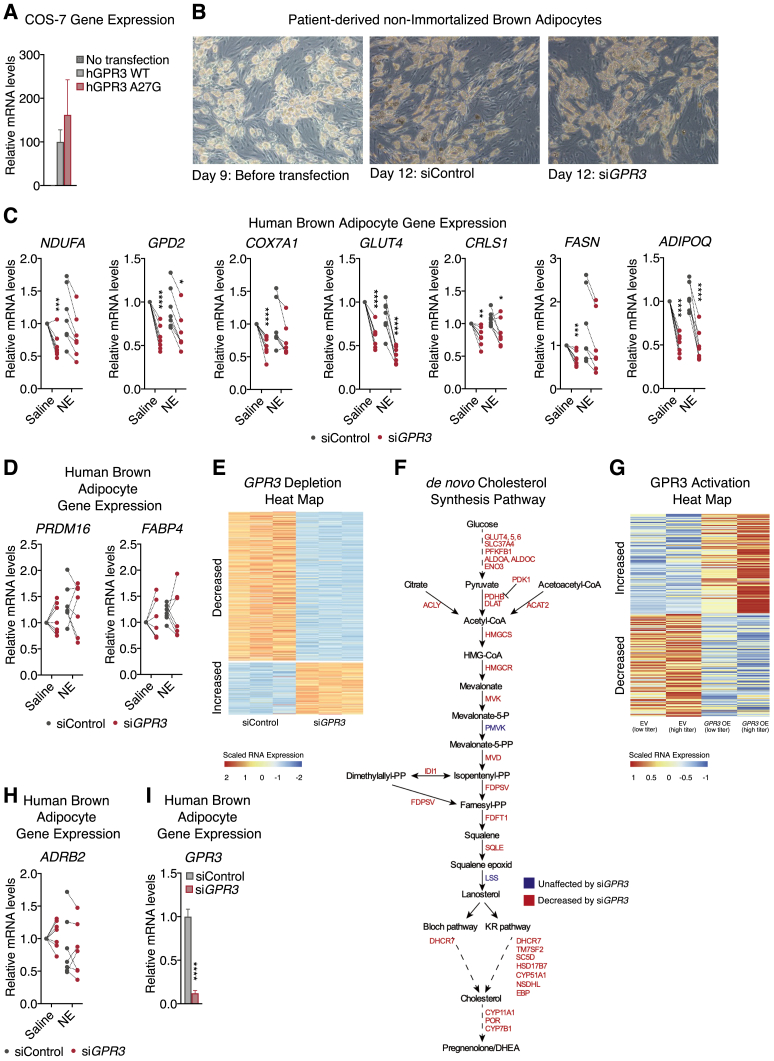
Characterization of GPR3 in human thermogenic adipocytes, related to Figure 7 (A) *GPR3* expression of transfected COS-7 cells for BRET-analysis. (B–D) (B) representative light microscopy images and, (C and D), gene expression of patient-derived, non-immortalized brown adipocytes following siRNA mediated *GPR3* knockdown and 4 hours vehicle or norepinephrine (NE) treatment. (E and F)) (E) heat map of gene regulation and, specifically, (F) genes in the *de novo* cholesterol synthesis pathway changed by *GPR3* depletion in patient-derived, non-immortalized brown adipocytes. (G) heat map of genes induced by GPR3 activation in patient-derived, non-immortalized brown adipocytes. (H) gene expression of patient-derived, non-immortalized brown adipocytes following siRNA mediated *GPR3* knockdown and 4 hours vehicle or NE treatment. (I) gene expression in human brown adipocytes following siRNA mediated *GPR3* knockdown. For all panels, error bars represent ±SEM, p ≤ 0.05 = ∗, p ≤ 0.01 = ∗∗, p ≤ 0.001 = ∗∗∗, p ≤ 0.0001 = ∗∗∗∗, t test.

We next evaluated the translational relevance of our murine studies of GPR3 regulation to humans. We identified a rare, disease-associated *GPR3* coding variant (MAF = 0.00051) in the Danish population that results in an alanine-to-glycine replacement (A27G) (Figure 7A). Interestingly, this mutation was located within the N-terminal region that we earlier identified as playing a role in intrinsic receptor activation (Figures 1F–1I). The A27G variant was associated with indications of lower whole-body insulin sensitivity (Figure 7A) and markedly decreased GPR3 constitutive Gs-signaling (Figures 7B and S6A). However, the ubiquitous impact of genetic polymorphism across all cells in the body limits the ability to infer tissue specific contributions of GPR3 in human adipose. Therefore, we measured *GPR3* expression in supraclavicular BAT from volunteers with a range of body mass indexes (BMI) and varying levels of glucose tolerance. Interestingly, higher levels of BAT *GPR3* were significantly associated with lower BMI in glucose tolerant individuals but not in donors with impaired glucose control (Figure 7C). These results link GPR3 in human BAT to systemic metabolic health.

To specifically address a causal role for GPR3 in human BAT biology, we acutely depleted *GPR3* in non-immortalized, supraclavicular brown adipocytes from seven donors (Figures 7D and S6B). *GPR3* knockdown in mature adipocytes from every donor dramatically reduced expression of *UCP1* and other thermogenic or metabolic genes under both basal and NE-stimulated conditions (Figures 7E and S6C) without impacting markers of differentiation (Figure S6D). Consistent with our murine transcriptomics studies, global interrogation of human GPR3-dependent gene programs revealed that loss of *GPR3* particularly impacted genes linked to lipid metabolism (Figures 7F and S6E). The gene program most significantly controlled by GPR3 was the mevalonate and cholesterol synthesis pathway (Figure S6F), which is a strong activator of human BAT (Balaz et al., 2019). These data suggest that GPR3 constitutive Gs-coupling shapes the thermogenic potential of human brown adipocytes even in the absence of adrenergic stimulation. In further agreement with our murine studies, dose-dependently increasing *GPR3* levels using lentivirus was fully sufficient to drive a global thermogenic gene signature (Figures 7G and S6G). The enrichments for fatty acid metabolic pathways and the response to dietary excess were especially complementary to the HFD-induced activation of GPR3 we observed in mice. Taken together, these gain and loss-of-function transcriptional signatures reveal the profound influence GPR3 has on human brown adipocyte thermogenic identity and activation.

**Figure S7:**
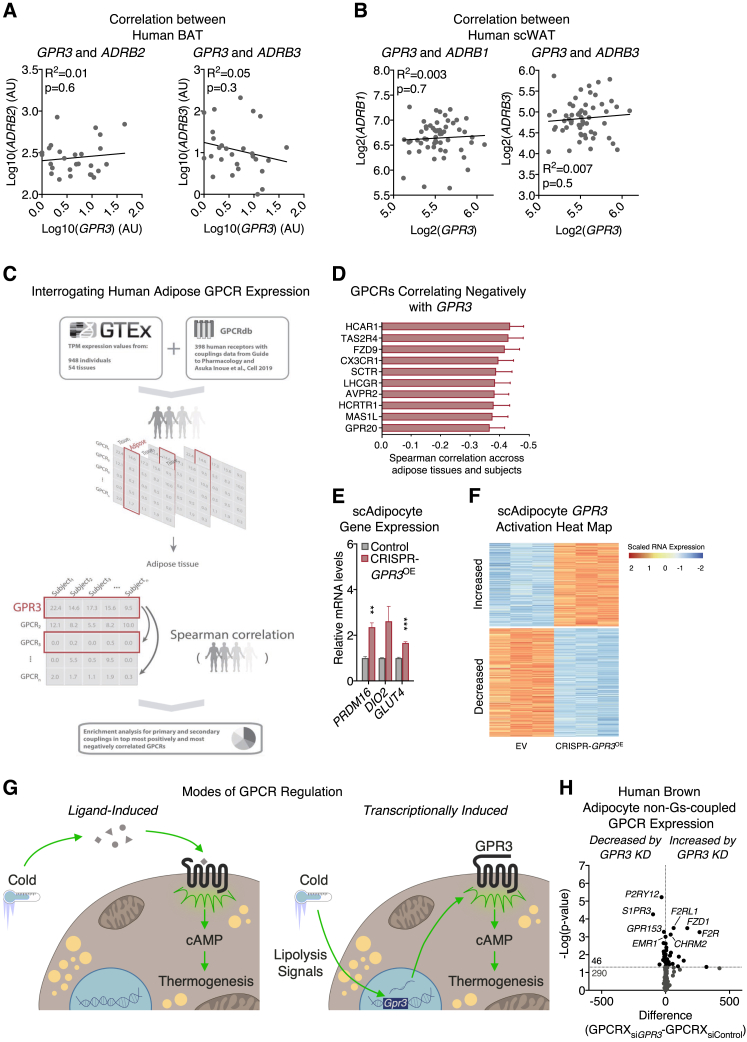
Characterization of GPR3 in human thermogenic adipocytes (continued), related to Figure 7 (A and B) Correlations between (A) *GPR3* and *ADRB2* as well as *GPR3* and *ADRB3* expression in human BAT and (B) *GPR3* and *ADRB1* as well as *GPR3* and *ADRB3* expression in human scWAT. (C) schematic depicting the analysis in GTEx of human GPCR co-regulation with *GPR3* (G protein-coupling data based on Inoue et al. 2019). (D) top ten GPCRs from the GTEx analysis that are negatively correlated with *GPR3*. (E and F) (E) gene expression and (F) heat map of global gene profiling of human subcutaneous white adipocytes with CRISPR-engineered *GPR3* overexpression. (G) model comparing the canonical ligand-based activation of GPCRs versus transcriptional induction of constitutively active receptors in the control of adipose thermogenesis. (H) change in the expression of 336 GPCRs, which do not primarily signal through Gs-coupling in human brown adipocytes following siRNA mediated *GPR3* knockdown. For all panels, error bars represent ±SEM, p ≤ 0.01 = ∗∗, p ≤ 0.001 = ∗∗∗, t test (E and H) or simple linear regression (A and B).

We next determined if a similar counter-regulation between *GPR3* and the β-adrenergic receptors existed in human adipocytes as that which we observed in mice (Figures 2A, 6J, S3D, S3E). We found that acute *GPR3* depletion in mature human brown adipocytes significantly increased the expression of *ADRB1* (Figures 7H and S6H). In fact, an unbiased assessment of the expression of all Gs-coupled receptors in human brown adipocytes revealed that *ADRB1* was the most significantly increased receptor following *GPR3* depletion (Figures 7I and S6I). This cellular counter-regulation was also observed in human thermogenic tissue where *GPR3* was negatively correlated with *ADRB1* in supraclavicular BAT biopsies (Figures 7J and S7A). Interaction between receptor expressions was not specific to BAT or *ADRB1*, because *GPR3* exhibited a significant negative correlation with *ADRB2* in human scWAT (Figures 7K and S7B). Collectively, our findings suggest an active regulatory network that adjusts GPCR expression to coordinate constitutive and inducible cAMP production and is conserved between mice and humans.

Given the *GPR3* counter-regulation with *ADRB2* observed in human scWAT, we next investigated a potential role for GPR3 in modulating thermogenic capacity of white adipose. *GPR3* expression in scWAT was positively correlated with *UCP1* (Figure 7L) in the same samples from the *ADRB2* analysis. Additionally, *GPR3* levels were significantly lower in scWAT from obese patients, where adipose lipolysis is diminished (Langin et al., 2005), but were normalized back to the levels of non-obese subjects following weight loss from bariatric surgery (Figure 7M). Further supporting the lipolytic control of *GPR3* in human WAT, we found that the GPCR most negatively correlated with *GPR3* in visceral and subcutaneous depots across 948 individuals (from the GTEx portal) was *HCAR1* (a.k.a. GPR81) (Figures S7C and S7D), a major regulator of adipose lipolysis (Ahmed et al., 2010).

To determine whether increasing *GPR3* could promote the thermogenic competence of subcutaneous white adipocytes, we employed CRISPR/Cas9 engineering to recruit the synergistic activation mediator complex (SAM) to the endogenous *GPR3* locus and drive expression. Raising *GPR3* levels in human subcutaneous white adipocytes induced thermogenic gene expression (Figures 7N and S7E), mitochondrial respiration (Figure 7O), and FA uptake (Figure 7P). Global transcriptional profiling revealed that gene programs linked to lipid metabolic processes and brown adipocyte differentiation were among the most significantly induced by GPR3 (Figures 7Q and S7F). Thus, boosting expression of *GPR3* is sufficient to elicit functional browning of human subcutaneous white adipocytes.

## Discussion

Activation of adipose thermogenesis is a dynamic, adaptive response (Cannon and Nedergaard, 2004; Klingenspor, 2003) orchestrated by numerous metabolite (Gnad et al., 2014), neuronal (Bartness et al., 2010; Razzoli et al., 2015), and hormonal signals (Beaudry et al., 2019b, 2019a; Collins, 2012; Li et al., 2018; Villarroya and Vidal-Puig, 2013; Villarroya et al., 2017) that converge on the surface of adipocytes. One common feature underlying these diverse regulatory means is that thermogenic activation is precipitated by the binding of a respective ligand to its cognate cell surface receptor. In this study, we uncover a parallel mechanism whereby cold exposure increases the expression of the constitutively active receptor, *Gpr3*, which possesses innate signaling capacity and, thus, can modulate cAMP levels and thermogenic output without a ligand (Figure S7G). We hypothesize that this high constitutive activity is why *Gpr3* expression must be kept at extremely low basal levels until there is a thermogenic demand. Mimicking the cold induction of *Gpr3* is then sufficient to drive and maintain elevated BAT activity even under conditions of little or no sympathetic tone. Collectively, our findings reveal that constitutive Gs-coupling by GPR3 cell-autonomously activates mouse and human thermogenic adipocytes and amplifies energy-expending signals in response to high fat diet.

Constitutive activity is not an exclusive feature of GPR3. GPR6 and GPR12 are members of the same subfamily of GPCRs with high basal Gs-coupled signaling capacity (Morales et al., 2018). In fact, over sixty GPCRs are reported to exhibit some degree of endogenous constitutive activity (Seifert and Wenzel-Seifert, 2002). Moreover, pathological human mutations have been found that confer intrinsic G protein signaling in receptors that are normally ligand-dependent (Lefkowitz et al., 1993). Yet, how receptors with natural innate activity are physiologically regulated is still largely unknown. Our findings that cold-triggered lipolytic signals induce *Gpr3* transcription provide an avenue of receptor control independent of external ligands. Conceivably, any lipolytic activator, adrenergic or nonadrenergic (Braun et al., 2018; Sahu et al., 2019; Villarroya and Vidal-Puig, 2013), would be able to invoke GPR3-dependent BAT thermogenesis.

Beyond GPR3 and adipose biology, the broader question remains as to why constitutively active receptors evolved at all. Presumably, this type of innate activity would provide cells in various tissues and physiological settings with the ability to set a basal cAMP tone to complement the more transient, ligand-induced spikes from other GPCRs. Our findings reveal an active coordination between the constitutive and ligand-induced cAMP signaling programs in mice and humans. This counter-regulation between Gs-coupled receptors suggests a system designed for defending a minimal cAMP signaling capacity while protecting against excess activation. The potential signaling compensation for reduced levels of GPR3 likely extends beyond Gs-coupled receptors as 46 out of 336 non-odorant receptors across all other G protein signaling modalities were significantly altered by *GPR3* depletion in human brown adipocytes (Figure S7H). These findings point toward a larger interconnected framework and orchestration of the GPCR-ome.

### Limitations of the study

One of the chief assumptions in this work is that transcriptional changes in GPCR expression directly reflect receptor levels at the cell surface. Future studies using tagging methods will be needed to determine the magnitude of changes occurring in cell surface occupancy. This type of analysis will be critical to fully delineate the mechanism by which the N-terminal region of GPR3 confers constitutive activity. Additionally, the question remains as to why lipolysis-induced *Gpr3* expression is not triggered in eWAT despite that tissue undergoing substantial lipolytic flux.

## STAR★Methods

### Key resources table

**Table:** 

REAGENT or RESOURCE	SOURCE	IDENTIFIER
**Antibodies**		

ATGL antibody	Cell Signaling	CAT#2138; RRID: AB_2167955
HSL antibody	Cell Signaling	CAT#4107; RRID: AB_2296900
GAPDH antibody	Cell Signaling	CAT#2118; RRID: AB_561053
Recombinant Anti-Tyrosine Hydroxylase antibody	Abcam	ab137869; RRID: AB_2801410
Donkey anti-Rabbit IgG (H+L) Highly Cross-Adsorbed Secondary Antibody, Alexa Fluor 568	Invitrogen	CAT#A10042; RRID: AB_2534017
Anti-UCP1 antibody	Thermo Scientific	N/A

**Biological samples**		

Human supraclavicular brown adipose tissue samples	Jespersen et al., 2020	N/A
Human subcutaneous adipose samples from non-obese and obese women.	Arner et al., 2012	N/A
Human subcutaneous adipose samples from non-obese (nonOB)/obese (OB)/post-obese (postOB) individuals.	Petrus et al., 2018	N/A
Patient-derived non-immortalized brown adipocyte culture	Jespersen et al., 2013	N/A

**Chemicals, peptides, and recombinant proteins**		

Tamoxifen (*in vivo* studies)	Sigma-Aldrich	CAT#T5648
CL-316,243	Sigma-Aldrich	CAT#C5976
Fungizone	Gibco	CAT#15290026
Gentamicin	Gibco	CAT#15710049
Zeocin	Gibco	CAT#R250-01
hBFGF	Sigma-Aldrich	CAT#F0291
Human insulin	Sigma-Aldrich	CAT#I9278
Dexamethasone (murine brown adipocyte culture)	Sigma-Aldrich	CAT#D4902
Dexamethasone (murine brown adipocyte culture, human brown adipocyte culture, CRISPR-*GPR3*^OE^ cell culture, *Gpr3* OE/wildtype murine primary adipocyte culture, patient-derived non-immortalized brown adipocyte culture)	Sigma-Aldrich	CAT#D1756
3,3′,5-Triiodo-L-thyronine sodium salt (T3)	Sigma-Aldrich	CAT#T6397
3-isobutyl-1-methylxanthine (IBMX)	Sigma-Aldrich	CAT#I5879
Rosiglitazone	Cayman Chemicals	CAT#71740
Fenofibrate	Cayman Chemicals	CAT#10005368
Cortisol	Sigma-Aldrich	CAT#H1035
Biotin	Sigma-Aldrich	CAT#B4639
D-Pantothenic acid hemicalcium salt	Sigma-Aldrich	CAT#P5155
Indomethacin	Sigma-Aldrich	CAT#I7378
4-Hydroxytamoxifen (*in vitro* studies)	Sigma-Aldrich	CAT#H6278
Primocin	Amaxa GmbH	CAT#VZA-1021
FGF-1	ImmunoTools	CAT#11343553
Holo-Transferrin human	Sigma-Aldrich	CAT#T0665
Polyethylenimine	Polysciences	CAT#23966-1
L-(−)-Norepinephrine (+)-bitartrate salt monohydrate	Sigma-Aldrich	CAT#A9512
(R)-(−)-Isoproterenol (adipocyte lipolytic activity, *ex vivo*)	Sigma-Aldrich	CAT#I6379
(−)-Isoproterenol hydrochloride (murine primary adipocyte gene expression)	Sigma-Aldrich	CAT#I6504
SR-3420	Rondini et al., 2017	N/A
Forskolin	Sigma-Aldrich	CAT#F6886
Atglistatin® (adipocyte lipolytic activity, *ex vivo*)	Mayer et al., 2013	N/A
Atglistatin (murine brown adipocyte and murine primary adipocyte gene expression)	Cayman Chemicals	CAT#15284
CAY10499	Cayman Chemicals	CAT#10007875
Triacsin C from Streptomyces sp.	Sigma-Aldrich	CAT#T4540
(R)-(+)-Etomoxir sodium salt	Tocris	CAT#4539
HSL inhibitor 76-0079	Novo Nordisk; Schweiger et al., 2006	N/A
Leupeptin	Roth	CAT#CN33.3
Antipain	Roth	CAT#2933.2
Pepstatin	Roth	CAT#2936.2
Sodium Pyruvate (100 mM)	Gibco	CAT#11360-070
Oligomycin A	Cayman Chemicals	CAT#11342
FCCP	Cayman Chemicals	CAT#15218
Antimycin A	Cayman Chemicals	N/A
Rotenone	Cayman Chemicals	CAT#13995
Chloroquine diphosphate salt	Sigma-Aldrich	CAT#C6628
Coelenterazine h	Invitrogen	CAT#C6780
18-27 peptide fragment sequence: NVNVSSVGPA.	This paper	N/A
Rodent Diet with 60 kcal% Fat	Research Diets	CAT#D12492
Rodent Diet with 10 kcal% Fat with 0.2% Fenofibrate	Research Diets	CAT#D15110904
Rodent Diet with 10 kcal% Fat with 0.005% Rosiglitazone Maleate	Research Diets	CAT#D15110905
60% kcal% fat HFD diet (site-directed adeno-associated virus (AAV) delivery)	Provimi Kliba SA	CAT#3436
DMEM - high glucose (murine brown adipocyte culture)	Sigma-Aldrich	CAT#D6429
DMEM (adipocyte respiration and adipocyte fatty acid (FA) uptake, *in vitro*)	Sigma-Aldrich	CAT#D5030
DMEM, high glucose, GlutaMAX™ Supplement, pyruvate (murine brown adipocyte culture, 293 FT cell culture, *Gpr3* OE/wildtype murine primary adipocyte culture)	Gibco	CAT#31966
DMEM, high glucose, GlutaMAX(TM) (HEK293-T cell culture)	Gibco	CAT#61965
Advanced DMEM/F-12 (human brown adipocyte culture)	Gibco	CAT#12634
DMEM, high glucose, L-Glutamine Supplement, pyruvate (CRISPR-*GPR3*^OE^ cell culture, 293 AAV cell culture)	Gibco	CAT#41965
DMEM 1885 (COS-7 cell culture)	Substrate department, UCPH	N/A
DMEM, high glucose, L-Glutamine, pyruvate (HEK293-T cell culture)	Gibco	CAT#11995
DMEM/F-12, GlutaMAX™ supplement (adipocyte lipolytic activity, *ex vivo*)	Gibco	CAT#31331
DMEM/F-12, HEPES, no phenol red (patient-derived non-immortalized brown adipocyte culture)	Gibco	CAT#11039
FluoroBrite™ DMEM	Gibco	CAT#A1896701
Opti-MEM I Reduced-Serum Medium (1X) (murine brown adipocyte transfection, human brown adipocyte transfection, patient-derived non-immortalized brown adipocyte transfection, site-directed lentiviral (LV) delivery)	Gibco	CAT#51985
Opti-MEM I Reduced Serum Medium (HEK293-T transfection, site-directed adeno-associated virus (AAV) delivery)	Gibco	CAT#31985
HBSS, calcium, magnesium (BRET-based miniG subtype recruitment assay)	Gibco	CAT#24020117
HBSS, calcium, magnesium, no phenol red (BRET-based cAMP sensing assay)	Gibco	CAT#14025092
Fetal Bovine Serum	Sigma-Aldrich	CAT#F7524
Fetal Bovine Serum (patient-derived non-immortalized brown adipocyte culture, 293 AAV cell culture)	Gibco	CAT#10270-106
Fetal Bovine Serum (HEK293-T cell culture)	Gibco	CAT#10500-064-500
Bovine Serum Albumin (*Gpr3* OE/wildtype murine primary adipocyte culture, adipose fractionation, *ex vivo*, IHC, adipocyte fatty acid (FA) uptake, *in vitro*)	Sigma-Aldrich	CAT#A7030
Bovine Serum Albumin (adipocyte lipolytic activity, *ex vivo*, immunoblotting)	Sigma-Aldrich	CAT#A6003
Bovine Serum Albumin (adipocyte lipolytic activity, *ex vivo*)	Roche	CAT#03117057001
Collagenase type I	Worthington Biochemical Corp.	CAT#LS004197
Collagenase type 2	Worthington Biochemical Corp.	CAT#LS004177
Collagenase D	Roche	CAT#11088882001
^18^F-FDG	Rigshospitalet, DK	N/A
Glycerol tri-3H-oleate-labeled triglyceride-rich lipoprotein (TRL)-mimicking particles	This paper	N/A
Deoxy-D-glucose, 2-[1,2-3H (N)]-	PerkinElmer	CAT#NET328A001MC
Ultima Gold	PerkinElmer	CAT#6013329
SOLVABLE	PerkinElmer	CAT#6NE9100
Perchloric acid (PCA)	Sigma-Aldrich	CAT#244252
GPCR qPCR array (mouse), Custom RT2 Profiler PCR Arrays	This paper	CAT#330171
GPCR qPCR array (human), Custom RT2 Profiler PCR Arrays	This paper	CAT#330171
ProLong™ Gold Antifade Mountant with DAPI	Invitrogen	CAT#P36931
Hematoxylin Solution, Mayer’s	Sigma-Aldrich	CAT#MHS32-1L
Eosin	Region Apoteket	CAT#856453
Bluing Buffer, Dako	Agilent	CAT#CS702
Pertex	Histolab	CAT#00840-05
Opal 690	Akoya Biosciences	CAT#FP1497001KT
Sudan black B	Sigma-Aldrich	CAT#199664
QIAzol Lysis Reagent	Qiagen	CAT#79306
Buffer RLT	Qiagen	CAT#79216
Lipofectamine™ RNAiMAX Transfection Reagent	Invitrogen	CAT#13778-150
Lipofectamine™ 2000 Transfection Reagent	Invitrogen	CAT#11668019
FuGENE® HD Transfection Reagent	Promega	CAT#E2311
Polybrene Infection/Transfection Reagent	Sigma-Aldrich	CAT#TR-1003
SYBER green Precision®PLUS qPCR Master Mix	Primerdesign	PPLUS-machine type
TaqMan® Fast Advanced Master Mix (2X)	Applied Biosystems	CAT#4444556
20X Taqman Assay Mix	Applied Biosystems	CAT#4331182 (Hs02330048_s1/Hs00240532_s1)
Maxima SYBR Green/ROX qPCR Master Mix (2X)	Thermo Scientific	CAT#K0223
Power SYBR™ Green PCR Master Mix	Applied Biosystems	CAT#4367659

**Critical commercial assays**		

High-Capacity cDNA Reverse Transcription Kit with RNase Inhibitor	Applied Biosystems	CAT#4374966
Transcriptor First Strand cDNA Synthesis Kit	Roche	CAT#04897030001
High-Capacity cDNA Reverse Transcription Kit	Applied Biosystems	CAT#4368814
RNeasy Mini Kit	Qiagen	CAT#74106
TruSeq RNA Library Prep Kit v2	Illumina	CAT#RS-122-2001/CAT#RS-122-2002
AAVanced Concentration Reagent	System Biosciences	CAT#AAV100A-1
HitHunter® cAMP Assay for Small Molecules	Eurofins DiscoverX	CAT#90-0075SM10
Pierce™ Detergent Compatible Bradford Assay Kit	Thermo Scientific	CAT#23246
Pierce™ BCA Protein Assay Kit	Thermo Scientific	CAT#23225
Clarity Western ECL Substrate	Bio-Rad	CAT#170-5061
RNAScope® Multiplex Fluorescent V2 Assay	Advanced Cell Diagnostics	CAT#446961 mm-Gpr3-01
Thermo Scientific™ Triglycerides Reagent	Thermo Fisher Scientific	CAT#TR22421
Free Glycerol Reagent	Sigma-Aldrich	CAT#F6428
Non-esterified free fatty acids NEFA HR-2 Assay Reagent	Wako Chemicals	CAT#436-91995
QBT Fatty Acid Uptake Assay Kit	Molecular Devices	CAT#R8132
Nano-Glo Luciferase Assay from Promega	Promega	CAT#N1110
QuikChange Site-Directed Mutagenesis Kit	Agilent Technologies	CAT#200518

**Deposited data**		

RNAseq C-3BO	This paper	#GSE173386
RNAseq hBA-OE	This paper	#GSE173404
RNAseq siRNAGPR3	This paper	#GSE173389
RNAseq hWA	This paper	#GSE173388

**Experimental models: cell lines**		

Murine brown preadipocytes	Harms et al., 2015	N/A
Human brown preadipocytes	Markussen et al., 2017	N/A
CRISPR-*GPR3*^OE^	This paper	N/A
COS-7	ATCC	CAT#CRL-1651
HEK293-T (site-directed lentiviral (LV) delivery)	ATCC	N/A
HEK293-T (BRET-based miniG subtype recruitment assay)	ATCC	N/A
293 AAV Cell Line	Cell biolabs	CAT#AAV-100
293 FT Cell Line	Invitrogen	CAT#R70007

**Experimental models: organisms/strains**		

C57BL/6NTac-Gt(ROSA)26Sor^tm2(CAG-Gpr3)Zpg^ (*Gpr3* TTG)	This paper	N/A
B6.FVB-Tg(Ucp1-cre)1Evdr/J	The Jackson Laboratories	JAX stock: 024670
B6-Tg(Ucp1-cre/ERT2)426Biat	Rosenwald et al., 2013	N/A
C-3KO	This paper	N/A
β-less	Bachman et al., 2002	N/A
Pnpla2^tm1Eek^, Tg(Adipoq-Cre)^1Evr^/J	Schreiber et al. 2017	N/A
Lipe^tm1Rze^/J	The Jackson Laboratories	JAX stock: 019004
C57BL/6NTac	Taconic Biosciences	N/A
C57BL/6NRj	Janvier Labs	N/A
B6129SF1/J	The Jackson Laboratories	JAX stock: 101043
C57BL/6N	Charles River Laboratories	N/A
C57BL/6J	Charles River Laboratories	N/A
B6.129-Gt(ROSA)26Sor^tm1(cre/ERT2)Tyj^/J	The Jackson Laboratories	JAX stock: 008463

**Oligonucleotides**		

Extensive list of primers, see Table S3	This paper	N/A
ON-TARGETplus SMARTPOOL: *Gpr3*	Dharmacon	CAT#L-045861-00-0005
ON-TARGETplus SMARTPOOL: *GPR3*	Dharmacon	CAT#L-003951-00-0005
ON-TARGETplus SMARTPOOL: *Lxra*	Dharmacon	CAT#L-040649-01-0005
ON-TARGETplus SMARTPOOL: *Lxrb*	Dharmacon	CAT#L-042839-00-0005
ON-TARGETplus Non-targeting Control Pool	Dharmacon	CAT#D-001810-10-20
siRNA targeting sequence: *Srebp1*	Sigma-Aldrich	SASI_Mm01_00135484
siRNA targeting sequence: *Pparg*	Sigma-Aldrich	SASI_Mm01_00172958
siRNA targeting sequence: *Ppara*	Sigma-Aldrich	SASI_Mm02_00319988
MISSION® siRNA Universal Negative Control #1	Sigma-Aldrich	CAT#SIC001
A27G mutation forward primer: 5′-cgtg ggcccaggagaggggccca-3′	This paper	N/A
A27G mutation reverse primer: 5′-tgggc ccctctcctgggcccacg-3′	This paper	N/A
h*GPR3* del 2-9 Forward: agcttg ccaccatggcctggctctcagc	This paper	N/A
h*GPR3* del 2-9 Reverse: gctga gagccaggccatggtggcaagct	This paper	N/A
h*GPR3* del 2-18 Forward: cttaagct tgccaccatggtgaatgtaagcagcgtg	This paper	N/A
h*GPR3* del 2-18 Reverse: cacgct gcttacattcaccatggtggcaagcttaag	This paper	N/A
h*GPR3* del 2-27 Forward: ctgtggg cccctccatggtggcaagc	This paper	N/A
h*GPR3* del 2-27 Reverse: gcttgcc accatggaggggcccacag	This paper	N/A
h*GPR3* del 2-36 Forward: ttcaagcttg ccaccatgctgccctcgcctaaggcc	This paper	N/A
h*GPR3* del 2-36 Reverse: ggccttagg cgagggcagcatggtggcaagcttgaa	This paper	N/A

**Recombinant DNA**		

lenti dCAS-VP64_Blast	Addgene	CAT#61425
lenti MS2-P65-HSF1_Hygro	Addgene	CAT#61426
lenti sgRNA(MS2)_zeo backbone	Addgene	CAT#61427
*GPR3* sgRNA: 5′ATGGG GGAGCGGGCGGTGCG-3′	This paper	N/A
Ucp1-*Adrb3* AAV	This paper	N/A
Ucp1-*Gfp* AAV	This paper	N/A
Ucp1-*Gpr3* AAV	This paper	N/A
*AAV* helper plasmid *pDP8*	Plasmid Factory	CAT#PF421-180518
pLenti CMV Puro DEST (w118-1)	Addgene	CAT#17452
pLenti CMV Puro DEST (w118-1) with *Gpr3* ORF	This paper	N/A
pLenti CMV Puro DEST (w118-1) with *Gfp* ORF	This paper	N/A
pLenti CMV Puro DEST (w118-1) with *GPR3* ORF	This paper	N/A
pLenti CMV Puro DEST (w118-1) with DRY-mutant *GPR3* ORF	This paper	N/A
pMDLg/pRRE	Addgene	CAT#12251
pRSV-Rev	Addgene	CAT#12253
pMD2.G	Addgene	CAT#12259
NES-NanoLuc-MiniG subtypes (miniGi, miniGs, miniGq, miniG12)	Professor Nevin Lambert, Augusta University, GA, USA	N/A
Plasma membrane marker Venus-Kras	Professor Nevin Lambert, Augusta University, GA, USA	N/A
pcDNA3.1(+) with *GPR3* ORF	This paper	N/A
pcDNA3.1(+) with DRY-mutant *GPR3* ORF	This paper	N/A
pcDNA3.1(+) with *CB1* ORF	This paper	N/A
YFP-Epac-RLuc (CAMYEL)	Jiang et al., 2007	N/A

**Software and algorithms**		

STAR	Dobin et al., 2013	N/A
HOMER	Heinz et al., 2010	N/A
iRNA-seq	Madsen et al., 2015	N/A
DESeq2	Love et al., 2014	N/A
Graphpad Prism 8.0 for statistical analysis	GraphPad	N/A

**Other**		

Phenomaster home cage system	TSE Systems	N/A
Constant climate chamber	Memmert	HPP750
Inveon multimodality PET/CT scanner	Siemens	N/A
Echo-MRITM-4in1 body composition analyzer	EchoMRI	N/A
EnVision multilabel plate reader	PerkinElmer	N/A
CLARIOstar plus	BMG Labtech	N/A
Synergy H1	BioTEK	N/A
Ultra-turrax homogenizer	IKA	N/A
ChemiDoc touch imaging system	Bio-Rad	N/A
Excelsior AS	Thermo Scientific	N/A
Kunz embedding centre	Kunz instruments	N/A
Microm ergostar HM 200	Marshall Scientific	N/A
Zeiss confocal microscope	Zeiss	LSM 700
HybEZ™ II hybridization system	Advanced Cell Diagnostics	N/A
Zeiss Axio observer microscope	Zeiss	N/A
LightCycler 480II	Roche	N/A
HiSeq 1500 system	Illumina	N/A
CONTOUR®NEXT EZ meter	CONTOUR®NEXT	N/A
Bayer contour next teststrimler	Bayer	CAT#84167836
Homeothermic monitor	Harvard Apparatus	N/A
HIDEX 300SL	HIDEX	N/A
TissueLyser II	Qiagen	85300
Seahorse XF96 cell culture microplates	Agilent Technologies	N/A
Seahorse XFe96 extracellular flux analyzer	Agilent Technologies	N/A
FlexStation 3 multi-mode microplate reader	Molecular Devices	N/A
PHERAstar microplate reader	BMG Labtech	N/A
Polyvinylidene fluoride (PVDF) transfer membrane	Roth	CAT#T830.1
Exome sequencing of 2,000 Danes	Lohmueller et al., 2013	LuCAMP consortia

### Resource availability

#### Lead contact

Further information and requests for resources and reagents should be directed to and will be fulfilled by the Lead Contact, Zachary Gerhart-Hines (zpg@sund.ku.dk).

#### Materials availability

Plasmids, arrays, and mouse models generated in this study will be available upon request.

#### Data and code availability

The accession number for data presented in Figures 4E–4G and Figure S4K [C3BO] is GEO: #GSE173386; the accession number for data presented in Figure 7F and Figures S6E and S6F [siRNAGPR3] is GEO: #GSE173389; the accession number for data presented in Figure 7G and Figure S6G [hBA-OE] is GEO: #GSE173404; the accession number for data presented in Figure 7Q and Figure S7F [hWA] is GEO: #GSE173388.

### Experimental model and subject details

#### Mouse models

All animal studies were performed with approved protocols from The Danish Animal Experiments Inspectorate (permit number: 2018-15-0201-01441) and the University of Copenhagen (project number: P18-312 and P19-374), with the exceptions of the PPAR agonist administration studies, the site-directed adeno-associated virus (AAV) and lentivirus (LV) delivery studies, and the studies performed on β-less mice and DAKO mice (see below). The following housing conditions apply to experiments that were carried out at the University of Copenhagen. Mice were housed in an enriched environment with *ad libitum* access to standard diet and tap water, unless otherwise stated. Light in the facility was set to a 12 h light/dark cycle (light: 6 AM-6 PM and dark: 6 PM-6 AM). Mice were housed at room temperature (RT=22±2°C) during breeding. All *in vivo* studies were performed in male animals. Mice were housed at thermoneutrality (TN=29±1°C) during conduction of experiments, unless otherwise stated. Mice were allowed to acclimatize to TN for 2-3 weeks prior to any experiment performed at TN. Acclimation was carried out in open cages in a constant climate chamber (Memmert, HPP750).

##### C-3BO

*Gpr3* targeted transgenic mice C57BL/6NTac-Gt(ROSA)26Sor^tm2(CAG-Gpr3)Zpg^ (*Gpr3* TTG) were generated by Taconic Biosciences. Constitutive BAT-specific overexpression of *Gpr3* (C-3BO) was obtained by crossing *Gpr3* TTG mice with B6.FVB-Tg(Ucp1-cre)1Evdr/J mice (JAX stock: 024670). Experimental animals were male C-3BO mice and control littermates (either heterozygous *Gpr3* TTG without the Cre transgene or Cre positive without the *Gpr3* TTG allele). The ages of experimental animals are reported for each individual study below.

##### I-3BO

Inducible BAT specific overexpression of *Gpr3* (I-3BO) was obtained by crossing *Gpr3* TTG mice (see above) with B6-Tg(Ucp1-cre/ERT2)426Biat mice (kindly provided by Professor Christian Wolfrum (ETHZ, CH)) (Rosenwald et al., 2013). Experimental animals were male I-3BO mice and control littermates (either heterozygous *Gpr3* TTG without the Cre transgene or Cre positive without the *Gpr3* TTG allele). Overexpression was induced by oral gavage of 2 mg tamoxifen (Sigma-Aldrich, T5648) in 100 μL corn oil once per day for 3 consecutive days. The ages of experimental animals are reported for each individual study below.

##### B-3KO

*Gpr3* conditional knockout mice (C-3KO) were generated by genOway. Constitutive BAT-specific knockout of *Gpr3* (B-3KO) was obtained by crossing C-3KO mice with B6.FVB-Tg(Ucp1-cre)1Evdr/J mice (JAX stock: 024670). Experimental animals were male B-3KO mice and control littermates (Cre negative mice expressing the floxed *Gpr3* allele). Unless otherwise stated, B-3KO mice and controls were housed at RT. The ages of experimental animals are reported for each individual study below.

##### β-less mice

β1, β2, β3-adrenergic receptor–knockout mice (β-less) maintained on a mixed background (FVB, C57BL/6J, and DBA) were developed as described previously (Bachman et al., 2002). Experimental animals were male β-less mice and control littermates of 3–4 months of age. Mice were group-housed in an enriched environment with *ad libitum* access to standard diet and water. Light in the facility was set to a 12 h light/dark cycle (light: 6 AM-6 PM and dark: 6 PM-6 AM). Unless otherwise stated, mice were housed at RT. Animals were maintained and cared for in accordance with the Guide for the Care and Use of Laboratory Animals (National Institutes of Health, Bethesda, MD, USA). Experimental procedures were approved by the University of Minnesota Animal Care and Use Committee.

##### DAKO mice

Double Adipocyte-specific ATGL and HSL Knock Out mice (DAKO) were obtained by crossing Pnpla2^tm1Eek^, Tg(Adipoq-Cre)^1Evr^/J mice (Schreiber et al., 2017) with Lipe^tm1Rze^/J mice. Mice were back-crossed for >10 generations on a pure C57BL/6J background. Experimental animals were male DAKO mice and control littermates of 16 weeks of age. Mice were group-housed in an enriched environment with *ad libitum* access to standard diet and water. Light in the facility was set to a 14 h light/10 h dark cycle (light: 6 AM-8 PM and dark: 8 PM-6 AM). Unless otherwise stated, mice were housed at RT. Animal protocols were approved by the Austrian Federal Ministry for Science, Research, and Economy (permit number: BMWF-66.007/0029-V/3b/2019) and the ethics committee of the University of Graz, and were conducted in compliance with the council of Europe Convention (ETS 123).

##### WT mice

The tissue panel presented in Figures 1J and S1C was performed on tissues isolated from C57BL/6NTac (Taconic Biosciences) mice. Experimental animals were male littermates of 20 weeks of age.

The GPCR qPCR array presented in Figures 1B, S1A, and S1B was performed on adipose tissues isolated from C57BL/6NTac (Taconic Biosciences) mice. Experimental animals were male littermates of 12 weeks of age.

The fractionation of interscapular BAT presented in Figure 1K was performed on adipose tissues isolated from C57BL/6NRj mice (Janvier Labs) mice. Experimental animals were male littermates of 12 weeks of age.

The CL-316,243 (Sigma-Aldrich, C5976) studies presented in Figures 4C–4G and S4H–S4L were performed on control littermates of I-3BO mice. Experimental animals were male mice of 15 weeks of age.

The PPAR agonist administration study presented in Figure 2K was performed on B6129SF1/J mice (JAX stock: 101043). Experimental animals were male littermates of 8-12 weeks of age. Mice were group-housed in an enriched environment with *ad libitum* access to standard diet and water, unless otherwise stated. Light in the facility was set to a 12 h light/dark cycle (light: 6 AM-6 PM and dark: 6 PM-6 AM). Mice were housed at RT. The study was approved by UPenn IACUC.

Site-directed adeno-associated virus (AAV) delivery studies were carried out in C57BL/6N mice (Charles River Laboratories). Experimental animals were male littermates of 5-6 weeks of age treated with AAV particles carrying *Gpr3*, *Adrb3*, or *Gfp* (procedure specified below). Mice were housed in individually ventilated cages in an enriched environment with *ad libitum* access to standard diet and water. Light in the facility was set to a reversed 12 h light/dark cycle (dark: 7 AM-7 PM and light: 7 PM-7 AM). Mice were housed at RT. Mice were single-housed 2 days prior to metabolic measurements using the Phenomaster Home Cage System (TSE Systems). The study was performed in accordance with FELASA guidelines and was approved by the Veterinary office of the Canton of Zürich.

Site-directed lentiviral (LV) delivery studies were carried out in C57BL/6J mice (The Jackson Laboratory). Experimental animals were male littermates of 8 weeks of age treated with LV particles either carrying *Gpr3* or *Gfp* (procedure specified below). Mice were housed in an enriched environment with *ad libitum* access to standard diet and water. Light in the facility was set to a 12 h light/dark cycle (light: 6 AM-6 PM and dark: 6 PM-6 AM). Mice were housed at RT. Mice were group-housed prior to transfer to the Phenomaster Home Cage System (TSE Systems). The study (Az.: 84-02.04.2016.A202) was performed in accordance with national guidelines and was approved by the Landesamt für Natur, Umwelt und Verbraucherschutz, Nordrhein-Westfalen, Germany.

#### Patient cohorts and samples

Human supraclavicular brown adipose tissue samples from normal glucose tolerant and impaired glucose tolerant individuals were characterized and described in detail in a separate manuscript, where a subset of the subjects was clinically characterized in depth and analyzed by RNA sequencing (Jespersen et al., 2020). Briefly, adipose biopsies were obtained from patients scheduled for surgery due to benign thyroid disease. Samples from patients (n=32) enrolled in the referenced “in depth” study were analyzed by RNA-sequencing. The data on GPR3 in relation to BMI and glucose tolerance were extracted and included in the present paper. One sample was removed based on poor RNA quality and excluded from the analysis. Human subcutaneous adipose samples from non-obese and obese women (n=56) have previously been described (Arner et al., 2012). Human subcutaneous adipose samples from non-obese (nonOB)/obese (OB)/post-obese (postOB) individuals for gene expression have previously been described (Petrus et al., 2018). The samples used in this study (n=15) were from the subgroup of cohorts 1, 2, and 3 which were globally transcriptionally profiled as reported. One outlier from each group was identified by the authors of the original report and was removed prior to the analysis in this current study. The disease association of the *GPR3* genetic variant was obtained from exome sequencing of 2,000 Danes in the previously described LuCAMP consortia (Lohmueller et al., 2013).

#### Cell lines

##### Murine brown adipocyte cell culture, Figures 2I and 2J

Murine brown preadipocytes were kindly provided by Associate Professor Patrick Seale (Harms et al., 2015). Preadipocytes immortalized with SV40 large T antigen were propagated in basal DMEM (Sigma-Aldrich, D6429) containing 10% FBS (Sigma-Aldrich, F7524) and 1% penicillin/streptomycin. Cells were passaged when they reached 70-80% of confluence. Media was changed every 2^nd^ day. Two days post 100% confluency, cells were induced to differentiate with DMEM supplemented 10% FBS, 1% penicillin/streptomycin, insulin (20 nM) (Sigma-Aldrich, I9278), dexamethasone (0.5 μM) (Sigma-Aldrich, D4902), T3 (1 nM) (Sigma-Aldrich, T6397), and 3-isobutyl-1-methylxanthine (IBMX) (0.5 mM) (Sigma-Aldrich, I5879). On day 2 of differentiation, dexamethasone and IBMX were omitted from the media. From day 4 of differentiation and onwards, cells were cultured in propagation medium. Cells were harvested or assayed on day 7 of differentiation. The cells were maintained at 37°C in a humidified atmosphere with 5% CO_2_.

##### Murine brown adipocyte cell culture, Figures 2L, 2M, 3A–3C, 6H–6J, and S2A

Murine brown preadipocytes were kindly provided by Associate Professor Patrick Seale (Harms et al., 2015). Preadipocytes immortalized with SV40 large T antigen were propagated in basal DMEM (Gibco, 31966) containing 10% FBS (Sigma-Aldrich, F7524) and 1% penicillin/streptomycin. Cells were passaged when they reached 70-80% of confluence. Media was changed every 2^nd^ day. On the day of 100% confluency, cells were induced to differentiate with DMEM supplemented 10% FBS, 1% penicillin/streptomycin, insulin (20 nM) (Sigma-Aldrich, I9278), dexamethasone (1 μM) (Sigma-Aldrich, D1756), rosiglitazone (0.5 μM) (Cayman Chemicals, 71740), T3 (1 nM) (Sigma-Aldrich, T6397), and 3-isobutyl-1-methylxanthine (IBMX) (0.5 mM) (Sigma-Aldrich, I5879). On day 2 of differentiation, dexamethasone, rosiglitazone, and IBMX were omitted from the media. Media was changed every 2^nd^ day. Cells were harvested or assayed on day 7 of differentiation. The cells were maintained at 37°C in a humidified atmosphere with 5% CO_2_.

##### Human brown adipocyte cell culture

Human brown preadipocytes were kindly provided by Associate Professor Jacob Bo Hansen (Markussen et al., 2017). Stromal-vascular cell fractions from deep human neck adipose tissue biopsies immortalized with human telomerase reverse transcriptase were propagated in Advanced DMEM/F12 (Gibco, 12634) containing 10% FBS (Sigma-Aldrich, F7524), 1% penicillin/streptomycin, L-glutamine (2 mM), Fungizone (250 μg/ml) (Gibco, 15290026), Gentamicin (10 mg/ml) (Gibco, 15710049), and human basic fibroblast growth factor (hBFGF) (2.5 ng/ml) (Sigma-Aldrich, F0291). Cells were passaged when they reached 70-80% of confluence. Media was changed every 2^nd^ day. On the day of 100% confluency, hBFGF was omitted from the media (designated day -2). On day 0, cells were induced to differentiate in Advanced DMEM/F12 supplemented with 2% FBS, 1% penicillin/streptomycin, L-glutamine (2 mM), insulin (5 μg/ml) (Sigma-Aldrich, I9278), dexamethasone (1 μM) (Sigma-Aldrich, D1756), 3-isobutyl-1-methylxanthine (IBMX) (0.5 mM) (Sigma-Aldrich, I5879), rosiglitazone (1 μM) (Cayman Chemicals, 71740), cortisol (1 μM) (Sigma-Aldrich, H1035), and T3 (1 nM) (Sigma-Aldrich, T6397). On day 3, the medium was refreshed with the same medium as used on day 0. On days 6, 9, and 12 of differentiation, IBMX, dexamethasone, insulin, and cortisol were omitted from the medium. Cells were harvested or assayed on day 12-14 of differentiation. The cells were maintained at 37°C in a humidified atmosphere with 5% CO_2_. This cell line was derived from cells isolated from a female subject.

##### CRISPR-*GPR3*^OE^ cell culture

CRISPR-*GPR3*^OE^ cells were kindly provided by Associate Professor Brice Emanuelli (Shamsi and Tseng, 2017; Xue et al., 2015). Cells were generated as previously described (Sustarsic et al., 2018). In brief, human fat stromal vascular fraction cells were induced to overexpress *GPR3* by lentiviral transduction of dCasp-VP64 (Addgene, 61425), MS2-P65-HSF1 (Addgene, 61426) (core-components of the CRISPRa-SAM system), and a *GPR3* sgRNA designed to target the *GPR3* promotor inserted into a sgRNA(MS2) backbone (Addgene, 61427). The *GPR3* sgRNA targeted the *GPR3* promoter at the -128 position upstream from the transcription start site with the following sequence: 5’ATGGGGGAGCGGGCGGTGCG-3’. sgRNA-expressing cells were selected using Zeocin (50 μg/mL) (Gibco, R250-01). CRISPR-*GPR3*^OE^ preadipocytes were cultured in DMEM (Gibco, 41965) supplemented with 10% FBS (Sigma-Aldrich, F7524) and 1% penicillin/streptomycin. Cells were passaged when they reached 70-80% of confluence. Media was changed every 2^nd^ day. On the day of 100% confluency, cells were induced to differentiate with DMEM supplemented with 10% FBS, 1% penicillin/streptomycin, biotin (33 μM) (Sigma-Aldrich, B4639), insulin (0.5 μM) (Sigma-Aldrich, I9278), pantothenate (17 μM) (Sigma-Aldrich, P5155), dexamethasone (0.1 μM) (Sigma-Aldrich, D1756), T3 (2 nM) (Sigma-Aldrich, T6397), 3-isobutyl-1-methylxanthine (IBMX) (500 μM) (Sigma-Aldrich, I5879), indomethacin (30 μM) (Sigma-Aldrich, I7378), rosiglitazone (1 μM) (Cayman Chemicals, 71740). Media was changed every 2^nd^ day. Cells were harvested or assayed on day 14 of differentiation. The cells were maintained at 37°C in a humidified atmosphere with 5% CO_2_. This cell line was derived from cells isolated from a male subject.

##### COS-7 cell culture

COS-7 cells (ATCC, #CRL-1651) were cultured in DMEM 1885 (Substrate department, UCPH) supplemented with 10% FBS (Sigma-Aldrich, F7524), 1% L-glutamine, and 1% penicillin/streptomycin. The cells were maintained at 37°C in a humidified atmosphere with 10% CO_2_.

##### HEK293-T cell culture (BRET-based miniG subtype recruitment assay)

HEK293-T cells (ATCC) were cultured in DMEM (Gibco, 11995) supplemented with 10% FBS (Gibco, 10500-064-500ml) and 1% penicillin/streptomycin. The cells were maintained at 37°C in a humidified atmosphere with 5% CO_2_.

##### HEK293-T cell culture (site-directed lentiviral (LV) delivery)

HEK293-T cells (ATCC) were cultured in DMEM (Gibco, 61965) supplemented with 10% FBS (Sigma-Aldrich, F7524) and 1% penicillin/streptomycin. The cells were maintained at 37°C in a humidified atmosphere with 3% CO_2_.

##### 293 AAV cell culture

293 AAV cells (Cell biolabs, AAV-100) were cultured in DMEM (Gibco, 41965) supplemented with 10% FBS (Gibco, 10270-106) and 1% penicillin/streptomycin. The cells were maintained at 37°C in a humidified atmosphere with 5% CO_2_.

##### 293 FT cell culture

293 FT cells (Invitrogen, R70007) were cultured in DMEM (Gibco, 31966) with 10% FBS (Sigma-Aldrich, F7524) and 1% penicillin/streptomycin. The cells were maintained at 37°C in a humidified atmosphere with 5% CO_2_.

#### Primary cell culture

##### Murine adipocytes

Whole-body inducible overexpression of *Gpr3* (*Gpr3* OE), used for primary cell studies, was obtained by crossing *Gpr3* TTG mice with B6.129-Gt(ROSA)26Sor^tm1(cre/ERT2)Tyj^/J mice (JAX stock: 008463). Experimental animals were *Gpr3* OE mice and control littermates (Cre positive without the *Gpr3* TTG allele). Male and female tissues were not pooled for generation of primary cell culture. BATs and scWATs were harvested from 4-6 weeks old control and *Gpr3* OE mice. Adipose tissues were isolated, minced, and subsequently digested with DMEM (Gibco, 31966) supplemented with 2% BSA (Sigma-Aldrich, A7030) and 0.2% collagenase type I (Worthington Biochemical Corp., LS004197) at 37°C (30-60 min). The digests were centrifuged (5 min, 400 g) to obtain the stromal vascular fraction (SVF) and pellets were resuspended in DMEM with 10% FBS (Sigma-Aldrich, F7524) and 1% penicillin/streptomycin. The cell suspensions were passed through a 40 μm strainer and distributed onto the plate-format of interest. On day 1 after cell harvest, the isolated preadipocytes were rinsed with DMEM containing 10% FBS and 1% penicillin/streptomycin and thereafter, media was changed every 2^nd^ day. The cells were passaged up to one time before differentiation. On the day of 100% confluency, cells were induced to differentiate with DMEM supplemented 10% FBS, 1% penicillin/streptomycin, insulin (86 nM) (Sigma-Aldrich, I9278), dexamethasone (0.1 μM) (Sigma-Aldrich, D1756), rosiglitazone (1 μM) (Cayman Chemicals, 71740), T3 (1 nM) (Sigma-Aldrich, T6397), and 3-isobutyl-1-methylxanthine (IBMX) (250 μM) (Sigma-Aldrich, I5879). On day 2 of differentiation, dexamethasone, rosiglitazone, and IBMX were omitted from the media. T3 was maintained in the media for BAT and omitted from the media for scWAT. Cells for *in vitro* fatty acid uptake studies and oxygen consumption measurements were re-plated on day 3 of differentiation (20,000-40,000 cells/well). *Gpr3* overexpression was induced by 4-OHT (1 μM) (Sigma-Aldrich, H6278) administration on day 5 and day 6 of differentiation. Both *Gpr3* OE cells and control cells were treated with 4-OHT. Cells were harvested or assayed on day 7 of differentiation. The cells were maintained at 37°C in a humidified atmosphere with 5% CO_2_. The *in vitro* fatty acid uptake and the gene expression panel studies presented in the paper were carried out on primary adipocyte cultures from female mice. The *in vitro* adipocyte respiration and the lipase inhibitor stimulation studies were carried out on primary adipocyte cultures from male mice. All experiments were repeated at least 3 times, and we did not observe any sex-depended differences in the physiological phenotype.

DAKO mice and control littermates used for primary cell culture were maintained as described above. scWAT was isolated from of 8-12 weeks old DAKO mice and control littermates. SVF cells were isolated as described above for *Gpr3* OE adipocytes with minor modifications: The digestion buffer contained 1.5 mg/ml collagenase D (corresponds to 0.285 IU/ml) (Roche, 11088882001), 3.2 mM CaCl_2_, 15 mM HEPES, and 0.5% BSA (Sigma-Aldrich, A6003). Isolated preadipocytes were maintained and propagated in DMEM/F12 media containing Glutamax (Gibco, 31331), 10% FCS, 1% penicillin/streptomycin, and primocin (Amaxa GmbH, VZA-1021). Prior to differentiation, SVF cells were seeded on 24-well plates and grown to confluency. On the day of 100% confluency, cells were induced to differentiate with DMEM supplemented with 10% FCS, 1% penicillin/streptomycin, insulin (0.87 μM), dexamethasone (1 μM), rosiglitazone (1 μM), and IBMX (0.5 mM). On day 2 of differentiation, dexamethasone, rosiglitazone, and IBMX were omitted from the media. Cells were assayed on day 8 of differentiation. The cells were maintained at 37°C in a humidified atmosphere with 7% CO_2_. All studies were carried out in adipocytes isolated from female animals.

##### Patient-derived non-immortalized brown adipocyte culture

Human supraclavicular BAT cells were kindly provided by Associate Professor Camilla Schéele (Jespersen et al., 2013). Human primary preadipocytes were cultured in DMEM/F-12 (Gibco, 11039) supplemented with 10% FBS (Gibco, 10270-106), 1% penicillin/streptomycin, and FGF-1 (ImmunoTools, 11343553). Cells were passaged when they reached 70-80% of confluence. Media was changed every 2^nd^ day. Two days past 100% confluency, cells were induced to differentiate with DMEM/F-12 supplemented with 1% penicillin/streptomycin, insulin (100nM) (Sigma-Aldrich, I9278), dexamethasone (0.1 μM) (Sigma-Aldrich, D1756), rosiglitazone (200 nM) (Cayman Chemicals, 71740), 3-isobutyl-1-methylxanthine (IBMX) (540 μM) (Sigma-Aldrich, I5879), T3 (2 nM) (Sigma-Aldrich, T6397), and transferrin (10 μg/mL) (Sigma-Aldrich, T0665). On day 3 of differentiation, media was changed and IBMX was omitted from the differentiation media. Thereafter, media was refreshed on days 6 and 9 and at both times, IBMX and rosiglitazone were omitted from the media. Cells were harvested or assayed on day 12 of differentiation. The cells were maintained at 37°C in a humidified atmosphere with 5% CO_2_. The siRNA mediated gene expression knockdown study was carried out on adipocyte cultures from male and female donors (M/F=4/3, age range=23-69). Adipocyte cultures isolated from a single donor (F, 26) were used for subsequent *GPR3* depletion and GPR3 activation and analyzed by RNA-sequencing.

### Method details

*In vivo* studies were not carried out under blinded conditions.

#### Metabolic phenotyping: High fat diet challenges

High fat diet (HFD) challenges were carried out by transition from normal chow diet to rodent diet with 60 kcal% fat (Research Diets, D12492), unless otherwise stated. Mice were group-housed unless relevant phenotyping strategies (indirect calorimetry) required single housing.

The acute HFD challenge of C-3BO mice and control littermates (n=5-7) presented in Figures 4A, 4B, 4E–4G, and S4G–S4L was carried out on 13-19 weeks old mice. Baseline energy expenditure measurements before transition to HFD are presented in Figure S4F. The animals were euthanized after 1-week HFD challenge and tissues were collected, weighed, and snap frozen for RNA isolation. In a separate study, 12-14 weeks old C-3BO mice and control littermates (n=4-6) were euthanized after 1-week HFD challenge and tissues were collected, weighed, and snap frozen for *in vivo* triglyceride-derived fatty acid uptake measurements (data presented in Figure 3M). Prior to the HFD challenge, mice had been monitored in the Phenomaster Home Cage System (TSE Systems) (data presented in Figures S4D and S4E).

The long-term HFD challenge of C-3BO mice and control littermates presented in Figures 5B–5G and S5A–S5E was initiated when mice (n=6) were 8-10 weeks of age. The glucose tolerance (GTT) of the animals was assessed following 8 weeks of HFD challenge. The study was repeated to test a side-by-side chow vs. HFD challenge (data presented in Figure 5A). For this study, one cohort of C-3BO mice and control littermates (n=2-7) was transitioned to HFD at 12-15 weeks of age, while an age-matched group of C-3BO mice and control littermates (n=5-10) was maintained on chow diet. The basal rodent phenotyping data presented in Figures 3L, S3F–S3I, and S4A–S4C were obtained from chow-fed mice (n=5-10) euthanized at 20-23 weeks of age.

To investigate the metabolic phenotype associated with daily administration of a beta-adrenergic agonist (data presented in Figures 4C–4G and S4H–S4L), mice (n=6) were exposed to daily injections of CL-316,243 (Sigma-Aldrich, C5976). The compound was administered intraperitoneally (1 mg/kg) every day (total 9 days) prior to the onset of dark phase. Mice were injected for 2 consecutive days prior to transition from chow diet to HFD and sacrificed after a 1-week HFD challenge. To facilitate an evaluation of chronic rather than acute effects of CL-316,243 administration, tissues were collected, weighed, and snap frozen for RNA isolation the day after the final CL-316,243 injection.

To investigate the metabolic phenotype resulting from acute induction of *Gpr3* overexpression in obese mice, tamoxifen (Sigma-Aldrich, T5648) was administrated in long-term HFD challenged I-3BO mice and control littermates (average bodyweight (BW)=42.7 g) (n=3-6) (data presented in Figures 5I, 5K, S5F, and S5H). The animals were euthanized 1 week after administration of the final dose of tamoxifen and tissues were collected, weighed, and snap frozen for RNA isolation. In a separate study, tamoxifen was administrated in long-term HFD challenged I-3BO mice and control littermates (average BW=49.3 g) (n=6). BWs were recorded 7 days after first dose of tamoxifen (data presented in Figures 5H and 5J). Finally, another cohort of obese I-3BO mice and control littermates (average BW=44.6 g) (n=6) were challenged with a GTT 5 weeks after tamoxifen administration (data presented in Figure 5N). This I-3BO cohort was housed at RT.

To investigate the energy expenditure profile of mice transitioned from chow diet to HFD following acute induction of *Gpr3* overexpression, tamoxifen was administered to I-3BO mice and control littermates (n=6) of 15-19 weeks of age before an HFD challenge was initiated as indicated in Figure S5G. The animals were euthanized after a 1-week HFD challenge and tissues were collected, weighed, and snap frozen for assessment of *in vivo* glucose uptake (data presented in Figure 5M). In a separate study, tamoxifen was administered to I-3BO mice and control littermates (n=5-6) of 18-19 weeks of age. The animals were immediately transitioned to HFD and euthanized after 1-week HFD challenge. Their tissues were collected, weighed, and snap frozen for assessment of *in vivo* triglyceride-derived fatty acid uptake (data presented in Figure 5L).

The acute HFD challenge of B-3KO mice and control littermates (n=6) presented in Figure 6G was carried out in 13-18 weeks old animals.

The long-term HFD challenge of B-3KO mice and control littermates presented in Figures 6A–6D was initiated when mice (n=6-7) were 11-13 weeks of age. The glucose tolerance (GTT) of the animals was assessed following 8 weeks of HFD challenge. The acute cold challenge was carried out after 9 weeks of HFD challenge. Mice were euthanized after 10-weeks of HFD challenge and tissues were collected, weighed, and snap frozen for RNA isolation.

The HFD challenge of AAV modified animals is elaborated in the designated section below.

#### Metabolic phenotyping: Cold challenges

To obtain tissue (n=6) and adipose fraction (n=5) resolution of cold-induction of *Gpr3*, single-housed mice were exposed to a 24 h cold challenge at 4°C. The cold challenges were carried out in open cages in a constant climate chamber (Memmert, HPP750) (data presented in Figures 1J, 1K, and S1C). Following the cold challenges, tissues were collected and snap frozen for RNA isolation.

The GPCR qPCR array presented in Figures 1B, S1A, and S1B was carried out on single-housed mice (n=3). To ensure cold exposure at the acute time points (3h and 8h), mice were transferred from their home cages to pre-chilled cages. For the remaining time points, mice were maintained in their home cages during the transition to cold. The cold challenge was carried out in open cages in a constant climate chamber (Memmert, HPP750) (4°C). After the cold challenge, tissues were collected and snap frozen for RNA isolation.

For the cold challenge of B-3KO mice presented in Figure 6E, a cohort of single-housed animals (n=4-5) aged 14-20 weeks was exposed to 4°C for 24 h before BATs were harvested, alongside an age-matched cohort (n=3-4) maintained at RT. The cold challenge was carried out in open cages in a constant climate chamber (Memmert, HPP750).

For the cold challenge of β-less mice presented in Figure 2A, a cohort of single-housed β-less mice and control littermates (n=6) were transferred inside their individual home cages to a refrigerated environmental enclosure (Columbus Instruments, OH) allowing precise control over the temperature within +/- 1°C. Mice were exposed to 4°C for 3 h before BATs were harvested, alongside an age-matched cohort (n=5-6) maintained at RT. The cold exposure was limited to 3 h because of the known cold intolerance of β-less mice (Bachman et al., 2002). After the cold challenge, BATs were collected and snap frozen for RNA isolation. Additionally, a cohort of group-housed β-less mice and control littermates (n=6) were housed at 30°C for four weeks to acclimate to thermoneutrality prior to euthanization, tissue collection, and RNA isolation (data presented in Figure S1G).

For the cold challenge of DAKO mice presented in Figure 2F, a cohort of single-housed animals (n=5-6) was exposed to 5°C for 6h before BATs were harvested alongside an age-matched cohort (n=4-5) maintained at RT. A separate cohort of single-housed DAKO mice and control littermates (n=3-4) were housed at 5°C for three weeks to acclimate to cold prior to euthanization (data presented in Figure 2G). The animals did not have access to enrichment during the cold challenges. BATs were collected and snap frozen for RNA isolation after the cold challenges. RNA was isolated as stated below using Trizol reagent combined with DNase treatment. cDNA was synthesized using High-Capacity cDNA Reverse Transcription Kit (Applied Biosystems, 4374966) and real-time quantitative PCR was carried out with Maxima SYBR Green/ROX qPCR Master Mix (2X) (Thermo Scientific, K0223).

#### The PPAR agonist administration

Rosiglitazone (Research Diets, D15110905) or fenofibrate (Research Diets, D15110904) was incorporated into low fat diets (LFD) at concentrations of 50 mg/kg of diet or 2 g/kg of diet, respectively. Animals (n=5) were fed for 2 weeks with either control LFD, rosiglitazone- or fenofibrate-containing LFD. Animals were euthanized at RT and BATs were collected and snap frozen for subsequent RNA isolation.

#### Site-directed adeno-associated virus (AAV) delivery

AAV plasmids were acquired from VectorBuilder. In brief, 10 μg of targeting vector plasmid was co-transfected with 40 μg of helper plasmid pDP8 (Plasmid Factory, PF421-180518) into 293 AAV cells (Cell biolabs, AAV-100) seeded at 60% confluence in a P15 culture flask using 200 μg Polyethylenimine (1 mg/ml) (Polysciences, 23966-1) in Opti-MEM (Gibco, 31985) according to the manufacturer’s protocol. The culture medium was refreshed 24 h post transfection. The culture medium was collected 72 h post transfection and concentrated using AAVanced Concentration Reagent (System Biosciences, #AAV100A-1) according to the manufacturer’s protocol.

For direct AAV mediated gene transfer into BAT, mice were injected as previously described (Modica et al., 2016). Mice were anesthetized with 1-3% isoflurane at an oxygen flow rate of 1L/min with VetFlo vaporizer. Body temperature was maintained using a closed loop heating pad. A 0.5-1.0 cm longitudinal incision at the interscapular region was performed to expose the brown fat depot. 10-20 μl of AAV (10^12^ vg/ml) were administered in both lobes of the brown fat depot by gentle insertion of about 1/4 of a 30G 8mm syringe needle. Following the injections, the incisions were sutured. After surgery, all mice received carprofen (5 mg/kg) by intraperitoneal injection. Animals were monitored daily.

4-5 weeks post operation, mice were transferred to cages compatible with the Phenomaster Home Cage System (TSE Systems). Mice were allowed 3 days to acclimate to the TSE cabinets at RT before transition to 60% HFD (Provimi Kliba SA, 3436). Animals were euthanized following 9 days of HFD challenge and tissues were collected and snap frozen for subsequent RNA isolation. Oxygen consumption and carbon dioxide production was measured every 15 min and the cabinet was set to a reversed 12 h light/dark cycle (dark: 7 AM-7 PM and light: 7 PM-7 AM). Indirect calorimetry data presented in this paper have not been normalized. Data are presented as smoothened values (average of 3 measurements before and 3 measurements after the data point). In presented figures, day/night reflects light/dark phases. Heat was calculated based on the formula by Weir (1949).

Total RNA was extracted from tissues using Trizol reagent according to the manufacturer’s instructions. DNase treatment was included, and cDNA was synthesized from 1 μg of RNA using the High Capacity cDNA Reverse transcription kit (Applied Biosystems, 4368814).

#### Site-directed lentiviral (LV) delivery

LV particles were obtained after calcium phosphate-based transfection of HEK293-T (ATCC) cells with Dest-e*Gfp*/Dest-*Gpr3* as previously described (Chen et al., 2013; Gnad et al., 2014). In brief, targeting vector plasmid as well as packaging plasmids pMDLg/pRRE (Addgene, 12251), RSV-rev (Addgene, 12253), and pMD2.G (Addgene, 12259) were co-transfected into HEK293-T cells seeded on Poly-L-lysine-coated 150-mm2 dishes. Medium was changed 16 h and 48 h post transfection. The supernatant was collected and centrifuged by an ultracentrifuge with SW32 Ti rotor at 61,700 *g* at 17°C for 2 h. Combined virus suspensions were concentrated by centrifugation over a 20% (w/v) sucrose cushion in a SW55 Ti rotor at 53,500 *g* at 17°C for 2 h.

For direct LV gene transfer into adipose tissue, mice were injected as previously described (Balkow et al., 2016). Mice were anesthetized with 3.5% isoflurane in O_2_ and maintained at 2% during the procedure. For the direct BAT injections, a small incision was performed in the interscapular area. 1000 ng of lentiviral particles carrying either *Gpr3* or *Gfp* were injected directly into each fat pad (Hamilton 861-01). Following the injections, the incisions were sutured. Mice were treated for post-operative pain by daily subcutaneous injections with carprofen 5 mg/kg after the operation and the following 3 days.

For acute metabolic phenotyping, mice were single-housed and transferred to cages compatible with the Phenomaster Home Cage System (TSE Systems) 72 h post operation. Here, mice were monitored for 48 h at 23°C. Animals were euthanized the following day and tissues were collected and snap frozen for subsequent RNA isolation. Oxygen consumption was measured every 18 min and the cabinet was set to a 12 h light/dark cycle (light: 6 AM-6 PM and dark: 6 PM-6 AM). The indirect calorimetry data have not been normalized. Data are presented as rolling averages (average of 5 measurements).

UCP1 staining was carried out as previously described (Gnad et al., 2014).

RNA was isolated as stated below with the exceptions that cDNA was synthesized from 500 ng RNA using Transcription First Strand cDNA Synthesis Kit (Roche, 04897030001) and real-time quantitative PCR was carried out with Power SYBR™ Green PCR Master Mix (Applied Biosystems, 4367659) using a QuantStudio 5 Real Time PCR System. Expression data were quantified by ΔC_T_ calculation and normalized to *Hprt*. Applied primers listed below:

**Table:** 

Transcript	Forward primer	Reverse primer
*Hprt*	GTCCCAGCGTCGTGATTAGC	TCATGACATCTCGAGCAAGTCTTT
*Ucp1*	TAAGCCGGCTGAGATCTTGT	GGCCTCTACGACTCAGTCCA

#### Unilateral denervation of interscapular BAT (iBAT)

iBAT of 20-21 weeks old C-3BO mice and control littermates (n=6) were unilaterally denervated as previously described (Fischer et al., 2019). Mice were housed at RT. The animals were anesthetized by inhalation of isoflurane (2.5% for induction, 1.5% for maintenance) and the incision site was shaved and disinfected using first 0.5% chlorohexidine in 85% ethanol and then 70% ethanol. Prior to surgery, mice received local anesthesia (lidocaine, 1.4 μg/g BW) and general analgesia (Rimadyl, 10 μg/g BW). The iBAT was prepared by a midline incision of the skin in the interscapular region and the detachment of the iBAT from the underlaying muscle layer. The five nerve fibers innervating the right BAT lobe were identified and cut (denervated) and the nerve fibers innervating the left BAT lobe were identified and touched with forceps (sham). Following the procedure, the fat pads were rinsed with sterile isotonic saline and the incision was closed with suture. The mice were individually housed in clean cages with access to a 37°C heating pad during the first 24 h post operation. Animals were monitored daily.

#### ^18^F-fluorodeoxyglucose (^18^F-FDG) PET/CT imaging

^18^F-FDG PET/CT Imaging was performed 1 week and 4 weeks after denervation surgery (described above). Mice were single-housed and maintained at RT. ^18^F-FDG (Rigshospitalet, DK) was administered intravenously between 9 AM and 2 PM. The average radioactive dose was 4.6 MBq (range: 3.5-5.7 MBq). Animals were fasted from 7 AM until the end of the imaging sessions on the days of imaging. Small animal PET/CT (Siemens, Inveon Multimodality PET/CT scanner) was performed 1 h after ^18^F-FDG administration. Mice were anaesthetized with sevoflurane 40 min after ^18^F-FDG injection until the end of the imaging session. PET data were acquired in list mode for 240 s and images were reconstructed using a 3-dimensional maximum a posteriori algorithm with CT-based attenuation and scatter correction. CT images were acquired using 360 projections, 65 kV, 500 mA, and 430 ms exposure and reconstructed with an isotropic voxel size of 0.210 mm. Images were analyzed using the Inveon software (Siemens). Quantitative analysis of the ^18^F-FDG uptake was performed by manually drawing regions of interest over the areas containing iBAT based on the CT images. Each iBAT lobe was separately quantified. The ^18^F-FDG uptake is expressed as % injected dose per gram tissue (%ID/g). Animals were euthanized after the second imaging session and the iBATs were isolated. Each iBAT lobe was cut in halves. One half was submerged in 4% formaldehyde (for histology) and the radioactivity measured by gamma counting (Wizard^2^, PerkinElmer). The other half was snap frozen for subsequent RNA isolation.

#### Mouse body composition analysis

Mouse lean and fat masses were determined by quantitative magnetic resonance (MR) using Echo-MRI™-4in1 Body Composition Analyzer (EchoMRI).

#### Glucose tolerance test (GTT)

Mice were fasted for 4 h prior to the conduction of GTTs. Glucose tolerance was assessed following an intraperitoneal injection of 2 g/kg lean body mass glucose solution in sterile water. Blood glucose was measured from tail vein blood samples collected just prior to injection (baseline) and 15, 30, 45, 60, 90, and 120 min post glucose injection. Blood glucose was measured using a CONTOUR®NEXT EZ meter and appropriate glucose indicator strips (Bayer, 84167836).

#### Acute cold challenge (rectal temperature measurements)

Mice had unlimited access to food and water during the experiment. After recording the baseline body temperature at RT, animals were transferred to new cold acclimated cages containing only bedding material for the duration of the 4 h cold challenge. At the end of the challenge, mice were returned to their home cages at RT. Core body temperature was obtained with a Homeothermic Monitor (Harvard Apparatus) by gently inserting a thermal probe into the mouse rectum.

#### Energy expenditure, *in vivo*

The following description applies to all indirect calorimetry data presented in the paper with the exception of the AAV studies and the LV studies (specified above).

The Phenomaster Home Cage System (TSE Systems) was employed for indirect calorimetry, food intake, and physical activity assessments. Mice acclimated to the TSE training cages for 5-7 days prior to transfer to the TSE cabinets and were allowed 2-3 days to acclimate to the TSE cabinets before baseline measurements, injections, or gavage. Oxygen consumption and carbon dioxide production were measured every 5 min and the cabinet was set to a 12 h light/dark cycle (light: 6 AM-6 PM and dark: 6 PM-6 AM). Indirect calorimetry data presented in this article have not been normalized. Data are presented as smoothened values (average of 6 measurements before and 6 measurements after the data point). Heat was calculated based on the formula by Weir (1949).

The norepinephrine (NE) challenge presented in Figure 6F was performed on 11-12 weeks old B-3KO mice maintained on chow diet. Mice were acclimated to RT and exposed to a 24h cold challenge (4°C) immediately before the NE-injection. The preceding cold challenge was carried out in a constant climate chamber (Memmert, HPP750). NE response was assessed in the TSE cabinet in anaesthetized mice (intraperitoneal injection of 75 mg/kg BW pentobarbital) following an intraperitoneal injection of 1 mg/kg BW NE (Sigma-Aldrich, A9512). The NE challenge was performed at TN and mice were sacrificed after the study.

#### Triglyceride (TG)-derived fatty acid (FA) uptake, *in vivo*

Glycerol tri-^3^H-oleate-labeled triglyceride-rich lipoprotein (TRL)-mimicking particles with an average diameter of 80 nm (representing large VLDL) were generated at Leiden University Medical Center as previously published (Khedoe et al., 2015).

Mice were fasted for 4 h prior to the conduction of the lipid clearance assay. 200 μL of radiolabeled TRL-mimicking emulsion particles (0.2 mg TG per mouse) were injected into the tail vein. Mice were maintained under a heating lamp during the procedure. To determine plasma decay of glycerol tri-^3^H-oleate, tail vein blood samples were collected into EDTA capillaries 2 min, 5 min, 10 min, and 15 min post injection. Capillaries were centrifuged (5 min, 5,867 *g*, 4°C). 5 μL of plasma was transferred into 2.5 mL of Ultima Gold (PerkinElmer, 6013329) and counted on HIDEX 300SL (HIDEX) for 10 min per sample. Plasma volumes were calculated as 0.04706 × BW (g).

Mice were euthanized by cervical dislocation 15 min post injection and perfused via the heart with ice-cold PBS (to remove non-internalized TRL-mimicking emulsion particles) before tissue collection. Tissue samples (50-100 mg) were dissolved O/N at 55°C in 1 mL SOLVABLE (PerkinElmer, 6NE9100). Dissolved tissues were transferred into 10 mL Ultima Gold and counted on HIDEX 300SL for 10 min per sample. Background counts (Scintillation fluid) were subtracted from each plasma or tissue sample counts and the output was recalibrated to account for the dilution factor. For tissue samples, relative dose uptake is presented as relative to tissue weight.

#### Glucose uptake, *in vivo*

Prior to conduction of the glucose clearance assay, mice were fasted for 4 h and anaesthetized for 30 min with 75 mg/kg BW pentobarbital. Mice were monitored on heating pads (37°C) during the procedure. 0.33 μCi/g BW ^3^H-labeled 2DG (2-deoxyglucose) (PerkinElmer, NET328A001MC) in 20% glucose solution was injected retro-orbitally to a final dose of 1 g/kg BW. To determine plasma decay of ^3^H, tail vein blood samples (25 μL) were collected just prior to injection (baseline) and 5 min, 10 min, 15 min, 20 min, and 25 min post injection. Additionally, blood glucose levels were measured at each timepoint using a CONTOUR®NEXT EZ meter and appropriate glucose indicator strips (Bayer, 84167836). Blood samples were immediately mixed with 60 μL BaOH. The solution was vortexed, and 60 μL of ZnSO_4_ was added to precipitate protein. Samples were centrifuged (5 min, 14000 g) and 50 μL of the supernatant was transferred into 3 mL of Ultima Gold (PerkinElmer, 6013329) and counted on HIDEX 300SL (HIDEX) for 10 min per sample.

Mice were euthanized by cervical dislocation 25 min post injection and tissues were collected. Tissue samples (10-100 mg) were homogenized in 800 μL ice cold lysis buffer (pH 7.4, 10% glycerol, 1% IGEPAL, 50 mmol/L HEPES, 150 mmol/L NaCl, 10 mmol/L NaF, 1 mmol/L EDTA, 1 mmol/L EGTA, 20 mmol/L sodium pyrophosphate, 2 mmol/L sodium orthovanadate, 5 mmol/L nicotinamide, 4 μmol/L thiamet G and protease inhibitors. 150 μL of the homogenate was deproteinized with 600 μL 4.5% perchloric acid (PCA). Another 150 μL of the homogenate was deproteinized with 300 μL BaOH and 300 μL ZnSO_4_ to precipitate 2-deoxyglucose-6-phosphate. Samples were vortexed and centrifuged (5 min, 14,000 *g*), 500 μL of the supernatant was transferred into 6 mL of Ultima Gold and counted on HIDEX 300SL for 10 min per sample.

Glucose uptake was calculated based on previously established methods (Ferré et al., 1985). Background blood 2DG count (baseline) was subtracted from each blood sample count and subsequently recalibrated to account for the blood dilution factor. AUC was calculated for both the glucose measurements (mg glucose) and the 2DG counts (timepoints: 0, 5, 10, 15, 20, 25 min), and a conversion factor was calculated (AUC: DPM/mg glucose). BaOH/ZnSO_4_-precipitation supernatant counts were subtracted from the Perchloric extract counts to obtain the tissue-trapped glucose and the result was recalibrated to account for the tissue dilution factor and normalized to tissue weight and the conversion factor. Glucose uptake is presented as ng/mg/25 min.

#### GPCR qPCR array

G protein-coupled receptor (GPCR) analysis was performed using customized qPCR plates (Qiagen, 330171) containing primers for 384 non-odorant GPCRs according to the manufacturer’s protocol. 44 Gs-coupled GPCRs were selected based on guidetopharmacology.org. Mouse expression data were normalized to *Rn18s*. Human expression data were normalized to the averaged expression of *ACTB, B2M, GAPDH,* and *YWHAZ.*

#### Quantification of BAT cAMP, *ex vivo*

cAMP was extracted from 10 mg of snap frozen iBAT. Tissues were homogenized in 200 μL of 5% trichloroacetic acid (TCA) in deionized water, using TissueLyser II (Qiagen, 85300) for 1 min at 30 Hz. Then, samples were centrifuged (10 min, 21,000 *g*, 4°C), allowing separation of the fatty layer. The fatty layer was removed before a second round of centrifugation (10 min, 21000 rpm, 4°C) and 150 μL of the transparent aqueous phase was isolated for subsequent analysis. Samples were brought to a volume of 800 μL with water-saturated ether, vortexed, and allowed for phase separation using a table centrifuge. The top ether layer was discarded. TCA extraction was repeated 4 times in total. Following the last extraction step, the residual ether layer was removed from the aqueous layer by heating the samples to 70°C. cAMP standards were prepared in deionized water. cAMP solutions were diluted 5-10x in deionized water before cAMP was measured using HitHunter® cAMP Assay for Small Molecules (Eurofins DiscoverX, #90-0075SM10) according to the manufacturer’s protocol. Luminescence was read on EnVision Multilabel Plate Reader (PerkinElmer). cAMP measurements were fitted using Sigmoidal, 4PL, X is log(concentration) fit line calculated with GraphPad Prism software. cAMP measurements were normalized to tissue weight.

#### Adipose fractionation, *ex vivo*

Mice were euthanized and BATs were isolated, minced, and subsequently digested with PBS supplemented with 5% BSA (Sigma-Aldrich, A7030), 4 mg/mL collagenase type I (Worthington Biochemical Corp., LS004197), and 10 mM CaCl_2_ at 37°C (20-30 min). The digests were passed through a 100 μm strainer and centrifuged (10 min, 400 *g*, 4°C). The floating adipocyte fraction and pelleted stromal vascular fraction were isolated for gene expression.

#### Quantification of liver triglycerides, *ex vivo*

Lipids were extracted from 50-200 mg of tissue by O/N incubation at 55°C in ethanolic KOH (66.6% 96% ethanol with 33.3% KOH). Samples were brought to a volume of 1,200 μL with 50% ethanol then centrifuged (5 min, maximum speed) in a microcentrifuge. After mixing 100 μL of supernatant with 100 μL of 0.5 M MgCl_2_, samples were kept on ice for 10 min. Samples were then centrifuged (5 min, maximum speed) in a microcentrifuge and supernatant was transferred to a new tube. Glycerol standards were prepared ranging from 1,000 mg/dL down to 1 mg/dL. In a standard 96-well plate, 3 μL of sample or standard was mixed with 300 μL of Thermo Scientific™ Triglycerides Reagent (Thermo Fisher Scientific, TR22421) and incubated at 37°C for 10 min before reading on CLARIOstar Plus plate reader (BMG Labtech). Triolein equivalents (TE) were fitted using a least squares (ordinary) fit line calculated with GraphPad Prism software. TE measurements were normalized to tissue weight.

#### Adipocyte lipolytic activity, *ex vivo*

To determine lipolytic activity in eWAT from β-less mice and control littermates, animals were fasted O/N and epidydimal fat pads were surgically excised in sterile conditions and digested using collagenase type 2 (Worthington Biomedical Corp., LS004177) in 1X Krebs-Ringer Solution (KRH) (120 mM NaCl, 5 mM KCl, 1.25 mM CaCl_2_, 0.5 mM MgCl_2_, 1.5 mM NaH_2_PO_4_, 0.7 mM Na_2_HPO_4_, 25 mM HEPES, 5.5 mM glucose) containing 1% BSA (Roche, #03117057001) (Cero et al., 2016). Each treatment condition was replicated in 2-4 separate experiments; for each experiment, fat pads from 4-5 mice were pooled and adipocytes incubated as described below. Each experiment consisted of 2-4 independent replicates. Total n varied from 4 to 25. Approximately 150,000-200,000 isolated adipocytes from pooled eWATs were incubated in KRH buffer + 4% BSA, 15 nM isoproterenol (ISO) (Sigma-Aldrich, I6379) or 10 μM forskolin (Fsk) (Sigma-Aldrich, F6886) for 180 min at 37°C with constant shaking at 140 rpm. Following the incubation period, lipolysis was assessed by measuring the release of free glycerol in the supernatant using Free Glycerol Reagent (Sigma-Aldrich, F6428) following the manufacturer’s instructions using a Synergy H1 plate reader (BioTEK). Glycerol content was normalized to total cellular protein content determined by Pierce™ Detergent Compatible Bradford Assay Kit (Thermo Scientific, #23246).

To determine lipolytic activity in scWAT from the DAKO mice, mature adipocytes differentiated from scWAT for 8 days (as described above) were incubated in DMEM/F12 media containing Glutamax (Gibco, 31331) supplemented with 2% BSA (Sigma-Aldrich, A6003) for 2 h. The supernatant was collected to assess basal lipolysis. For lipase inhibition, cells were pretreated with 40 μM Atglistatin® (Mayer et al., 2013) and 10 μM HSL inhibitor 76-0079 (Novo Nordisk) (Schweiger et al., 2006) for 30 min. Then, media was changed and adipocyte lipolysis was stimulated in the absence or presence of 40 μM Atglistatin/10 μM 76-0079 using 10 μM isoproterenol (ISO) (Sigma-Aldrich, I6379) for 60 min. The supernatant was collected to assess ISO-stimulated lipolysis. Lipolysis was measured as the release of fatty acids into the media using Non-esterified free fatty acids NEFA HR-2 Assay Reagent 2 (Wako Chemicals, 436-91995) following the manufacturer’s instructions. fatty acid content was normalized to total cellular protein content determined using Pierce™ BCA Protein Assay Kit (Thermo Scientific, 23225) with BSA (Sigma-Aldrich, A6003) as standard.

#### Immunoblotting

BAT depots of DAKO mice and control littermates were homogenized in ice-cold solution A (0.25 M sucrose, 1 mM EDTA, 1 mM dithiothreitol, pH: 7.0 supplemented with 20 mg/ml leupeptin (Roth, CN33.3), 2 mg/ml antipain (Roth, 2933.2), 1 mg/ml pepstatin (Roth, 2936.2)) using Ultra-Turrax Homogenizer (IKA). Homogenates were centrifuged (10 min, 1,000 g, 4°C) and an aliquot of the supernatant including the fatty layer was delipidated O/N at -20°C using a 5-fold volume of ice-cold acetone. Proteins were precipitated by centrifugation (30 min, 20,000 *g*, 4°C) and the protein pellet was solubilized in 0.3 N NaOH and 0.1% SDS at 56°C. Protein concentrations were determined using Pierce BCA Protein assay (Thermo Scientific, 23225) with BSA (Sigma-Aldrich, A6003) as standard. For immunoblotting, an aliquot of BAT homogenate containing 10 μg of protein was delipidated and precipitated as described above. Proteins were denatured in SDS sample buffer, resolved using a 10% SDS-PAGE, and transferred onto a polyvinylidene fluoride (PVDF) transfer membrane (Roth, T830.1) in CAPS buffer (10 mM CAPS, 10% methanol, pH: 11.0). The membrane was blocked with 10% blotting grade milk powder in TST (50 mM Tris-HCl, 0.15 M NaCl, 0.1% Tween-20, pH: 7.4). The membrane was incubated O/N at 10°C with primary antibodies for ATGL (Cell Signaling, #2138), HSL (Cell Signaling, #4107), and GAPDH (Cell Signaling, #2118). Protein expression was visualized using anti-rabbit-HRP and enhanced chemiluminescence using Clarity Western ECL Substrate (Bio-Rad, 170-5061) and ChemiDoc Touch Imaging System (Bio-Rad). The presented data have not been quantified.

#### Histology and immunofluorescence staining

With the exception of UCP1 staining of BAT following site-directed LV delivery of *Gpr3* (see above), all histology and immunofluorescence staining was carried out as stated below.

The immunofluorescence protocol was modified from Fischer et al. (2019). iBATs were isolated and fixed in 4% formaldehyde at 4°C for 3 days. The tissues were automatically dehydrated using Excelsior AS (Thermo Scientific) and embedded in paraffin using Kunz embedding centre (Kunz instruments). Subsequently, 5 μm sections were prepared on Microm Ergostar HM 200 (Marshall Scientific) and dried on glass slides O/N at 37°C. The sections were rehydrated with the following steps: 3 × 10 min in Xylene, 3 × 5 min in absolute ethanol, 2 × 5 min in 96% ethanol, 5 min in 70% ethanol, 5 min in PBS + 0.1% Triton X-100. Subsequently, slides were boiled for 30 min in citrate buffer (pH 6) for antigen retrieval. When slides had cooled to RT, background fluorescence was blocked by 5 min incubation in 0.1% Sudan black B (Sigma-Aldrich, 199664) in 70% ethanol. Slides were rinsed in 70% ethanol and transferred to PBS + 0.1% Triton X-100. Blocking was performed with PBS + 0.1% Triton X-100 + 3% BSA (Sigma-Aldrich, A7030) for 1 h at RT. Anti-Tyrosine Hydroxylase antibody (Abcam, ab137869) was diluted in PBS + 0.1% Triton X-100 + 3% BSA (1:200) and applied onto the slides for incubation in a humidified chamber O/N. The following day, slides were washed 3 × 10 min in PBS + 0.1% Triton X-100 and incubated for 1 h at RT with secondary antibody (Donkey anti-Rabbit IgG (H+L) Highly Cross-Adsorbed Secondary Antibody, Alexa Fluor 568) (Invitrogen, A10042) diluted in PBS + 0.1% Triton X-100 + 3% BSA (1:800). Subsequently, slides were washed 3 × 10 min in PBS + 0.1% Triton X-100 and mounted using ProLong™ Gold Antifade Mountant with DAPI (Invitrogen, P36931). Images were acquired on a Zeiss confocal microscope (LSM 700) using Zen software.

For hematoxylin and eosin staining, sections were prepared and rehydrated as stated above. Sections were stained in filtered 50% hematoxylin in water (Sigma-Aldrich, MHS32-1L) for 10 min, rinsed in tap water, incubated in bluing buffer (Agilent, CS702) for 2 min, rinsed in tap water, stained in eosin (0.1% solution in Walpole’s acetate buffer 0.1 M, pH=4.6 (Region Apoteket, 856453)) for 30 sec, rinsed in tap water, and dehydrated in 96% ethanol, 100% ethanol, and 2 × 5 min in Xylene. Finally, sections were embedded in Pertex (Histolab, 00840-05). The presented data have not been quantified.

#### *In situ* hybridization (ISH)

Sections for RNAscope ISH were formalin-fixed and paraffin-embedded as stated above. ISH was performed using RNAScope® Multiplex Fluorescent V2 Assay (Advanced Cell Diagnostics, mm-Gpr3-01 #446961) with a HybEZ™ II Hybridization System (Advanced Cell Diagnostics) and standard pretreatment and hybridization conditions according to the manufacturer’s instructions. Opal 690 (Akoya Biosciences, FP1497001KT) at 1:1000 dilution was used for signal visualization. Images were acquired using Zeiss Axio Observer microscope (Zeiss) with Axiocam 702 camera. The presented data have not been quantified.

#### Gene expression analysis (RT qPCR and RNA-sequencing)

Gene expression analysis was carried out as described below unless otherwise specified.

Tissues were isolated and immediately snap frozen in liquid nitrogen. Cells were lysed using QIAzol Lysis Reagent (Qiagen, 79306) or buffer RLT (Qiagen, 79216). RNA isolation was performed using RNeasy Mini Kit (Qiagen, 74106) or manual purification according to the manufacturer’s protocol (Qiagen, 79306). Because the *Gpr3* ORF consists of a single exon, DNase treatment was included to ensure accurate detection. cDNA synthesis was carried out on 500–1000 ng RNA using High Capacity cDNA Reverse Transcription kit (Applied Biosystems, 4368814). cDNA synthesis was performed on Mastercycler pro (Eppendorf) according to the manufacturer’s protocol. With the exceptions of *ADRB1* and *ADRB2,* gene expression levels were quantified using SYBER green Precision®PLUS qPCR Master Mix (Primerdesign, PPLUS-machine type)-based real-time quantitative PCR using LightCycler 480II (Roche) according to the manufacturer’s protocol. A complete list of applied primers is available in Table S3. *ADRB1* and *ADRB2* gene expression levels were quantified using 20X Taqman Assay Mix (Applied Biosystems, 4331182 (Hs02330048_s1/Hs00240532_s1)) and TaqMan® Fast Advanced Master Mix (2X) (Applied Biosystems, 4444556) according to the manufacturer’s protocol. Expression data were quantified by ΔC_T_ calculation normalized to *36b4/36B4* (Rplp0/RPLP0), unless otherwise stated, and is presented as 2ˆΔC_T_ or relative expression compared to a defined control group. The *Gpr3* (ORF) primer set was applied for quantification of transgenic overexpression and loss-of-function of *Gpr3* while the *Gpr3* (UTR) primer set was applied for quantification of endogenous expression of *Gpr3*.

For generation of RNA-sequencing libraries, polyadenylated mRNA was isolated from 1 μg of total RNA by incubation with oligo-dT beads and prepared according to the manufacturer’s protocol using TruSeq RNA Library Prep Kit v2 (Illumina, RS-122-2001/ RS-122-2002). Samples were sequenced using the HiSeq 1500 System (Illumina). Sequencing reads were mapped to the mouse or human reference genome (version mm9 or hg19) using STAR (Dobin et al., 2013). Tag directories were generated using HOMER (Heinz et al., 2010) and exon reads were counted using iRNA-seq (Madsen et al., 2015). Normalization and identification of differentially expressed genes was performed using DESeq2 (Love et al., 2014). RNA-sequencing have been deposited in GEO (GEO: GSE173390). Pathway analysis was performed using GO enrichment analysis. Presented pathways have a P<0.05 (test type: Fisher’s Exact and correction: FDR). For mouse tissue analysis, the applied Log2FC criteria was Log2FC>1 and Log2FC<-1. For human cell analysis, the applied Log2FC criteria was Log2FC>0.5 and Log2FC<-0.5 (with the exception of GPR3 activation in patient-derived non-immortalized brown adipocytes. Here the applied Log2FC criteria was Log2FC>0.6 and Log2FC<-0.6). Pathways are sorted based on fold enrichment (over expected enrichment with n numbers of genes). For the pathway enrichment analysis of GPR3 activation in patient-derived non-immortalized brown adipocytes, only the high titer (n=1) was included.

#### Stimulation of murine brown adipocytes, *in vitro*

Lipase inhibitor, Triascin C, Etomoxir treatment: On day 7 of differentiation, media was aspirated and replaced with maintenance media supplemented with lipase inhibitors (10 μM Atglistatin (Cayman Chemicals, 15284) and 20 μM CAY10499 compound (Cayman Chemicals, 10007875) or 5 μM Triascin C (Sigma-Aldrich, T4540) or 50 μM Etomoxir (Tocris, 4539). After 1 h preincubation, cells were stimulated with 100 nM isoproterenol (ISO) (Sigma-Aldrich, I6504) or 20 μM SR-3420 (Rondini et al., 2017). Cells were harvested for RNA extraction after 3 h stimulation.

NE stimulation: On day 7 of differentiation, cells were stimulated with 1 μM NE (Sigma Aldrich, A9512) or sterile H_2_O. Cells were harvested for RNA extraction after 1 h stimulation.

#### Stimulation of patient-derived non-immortalized brown adipocytes, *in vitro*

NE stimulation: On day 12 of differentiation, media was aspirated and replaced DMEM/F-12 (Gibco, 11039) supplemented with 1% penicillin/streptomycin. After 2 h preincubation, cells were stimulated with 10 μM NE (Sigma Aldrich, A9512) or sterile H_2_O. Cells were harvested for RNA extraction after 4 h stimulation.

#### siRNA mediated gene expression knockdown

Immortalized mouse and human brown preadipocytes were differentiated as stated above. On day 3/9 (mouse/human), of differentiation, cells were reverse transfected according to Isidor et al. (2015). In brief, siRNA targeting the gene of interest or relevant control siRNA were diluted in Opti-MEM (Gibco, 51985) to a final concentration of 50 nM. A complete list of applied siRNAs and relevant controls are available in the KRT. In a separate tube, RNAiMAX (Invitrogen, 13778-150) was diluted in Opti-MEM to a final concentration of 5 μL/ml. The diluted siRNA was added to the RNAiMAX solution 1:1. The siRNA mix was added to the bottom of the wells in the plate-format of interest and allowed to incubate at RT for 30 min. In the meantime, cells were trypsinized, counted, and resuspended in culture media. Finally, the cell suspension was distributed on top of the siRNA mix to a final siRNA concentration of 5 nM. Media was changed 2/3 days after transfection and cells were harvested or assayed on day 7/12 of differentiation.

Patient-derived non-immortalized brown adipocytes were differentiated as stated above. On day 9 of differentiation, cells were forward transfected (20 nM of siRNA). Forward transfection was carried out using RNAiMAX, Opti-MEM, and siRNA targeting *GPR3* (Dharmacon, L-003951-00-0005) or control siRNA (Dharmacon, D-001810-10-20) according to the manufacturer’s protocol. Cells were harvested on day 12 of differentiation.

#### Lentiviral (LV) delivery, *in vitro*

293 FT (Invitrogen, R70007) cells were used for lentivirus production. The day before transfection, 293 FT cells were plated onto a 6-well plate at a density of 3.5 × 10ˆ6 cells/well. The following day, cells were transfected with (per well) 1 μg pMD2.G (Addgene, 12259), 2 μg pMDLg/pRRE (Addgene, 12251), 1 μg pRSV-Rev (Addgene, 12253), and 4 μg custom pLenti CMV Puro DEST (w118-1) carrying either the *GPR3* coding sequence (NM_005281.4) (Genscript) or the DRY-mutant *GPR3* (Genscript) or EV (Addgene, 17452). In a separate tube, 24 μL FuGENE HD (Promega, E2311) was diluted in 776 μL Opti-MEM (Gibco, 51985), vortexed briefly and allowed to incubate for 5 min before the DNA solution was added. Transfection mixes incubated at RT for 20 min before they were dropwise distributed onto the 293 FT cells. Virus-containing supernatant was harvested and sterile filtered 48 h post transfection. Virus-containing supernatant was stored in -80°C until transduction.

Forward transduction: Mouse brown preadipocytes were differentiated as stated above. On day 7 of differentiation, cells were forward transduced with serial dilutions of sterile-filtered virus-containing supernatant (0.5%, 1%, 5%, 10%) in culture medium + 0.1% polybrene (Sigma-Aldrich, TR-1003). For the studies presented in Figures 3A–3C, cells incubated in media containing 5% sterile-filtered virus-containing supernatant. Cells were harvested or assayed on day 10 of differentiation.

Patient-derived non-immortalized brown adipocytes were differentiated as stated above. On day 9 of differentiation, cells were forward transduced with dilutions of sterile-filtered virus-containing supernatant (6.66%, 33.33%) in culture medium + 0.1% polybrene. Cells were harvested on day 12 of differentiation.

#### Adipocyte respiration, *in vitro*

Cells were cultured on Seahorse XF96 Cell Culture Microplates (Agilent Technologies). Cell culture medium was changed 1 h before the first measurement to DMEM (Sigma-Aldrich, D5030) supplemented with 25 mM glucose and 1 mM pyruvate (Gibco, 11360-070). Real-time oxygen consumption rate (OCR) was measured under basal conditions and following injections of oligomycin A (1 μM) (leak respiration) (Cayman Chemicals, 11342), NE (1 μM) (Sigma-Aldrich, A9512) (NE-stimulated respiration), FCCP (0.5 μM) (Cayman Chemicals, 15218) (maximal respiratory capacity), antimycin A/rotenone (1 μM each) (Cayman Chemicals, N/A) (Cayman Chemicals, 13995) (non-mitochondrial respiration). OCR was assessed using a Seahorse XFe96 Extracellular Flux Analyzer (Agilent Technologies). The presented values represent the raw reads and have not been normalized. The quantification for statistical analysis represents the values of the final measurement for each drug administration with the exception of NE-stimulated respiration (NE-stim.) which represent the delta between the leak respiration and maximal NE-induced OCR.

#### Adipocyte fatty acid (FA) uptake, *in vitro*

Cells were cultured in 96-well assay plates with black edges and clear bottom. Cell culture medium was changed 1 h before the first measurement to DMEM (Sigma-Aldrich, D5030) supplemented with 5 mM glucose and 1 mM pyruvate (Gibco, 11360-070). Immediately before adding the loading buffer, cells were stimulated with NE (1 μM) (Sigma-Aldrich, A9512). Loading buffer (2% HEPES buffer + 0.2% BSA (Sigma-Aldrich, A7030) in PBS) was mixed with Fatty Acid Uptake Assay Reagent Component A (QBT Fatty Acid Uptake Assay Kit) (Molecular Devices, #R8132) and distributed onto the cells. Immediately after, real-time fatty acid uptake was assessed using FlexStation 3 Multi-Mode Microplate Reader (Molecular Devices) for 1 h. Fatty acid uptake as presented in arbitrary units (AU) represent the raw reads and has not been normalized. The quantification for statistical analysis represents the values of the final measurement.

#### Bioluminescence resonance energy transfer (BRET)-based cAMP sensing

cAMP production was measured with a BRET-based cAMP assay applying the cAMP sensor YFP-Epac-RLuc (CAMYEL) (Jiang et al., 2007) in live COS-7 cells. A27G mutation was introduced in the *GPR3* coding sequence (NM_005281.4) in a custom pcDNA3.1(+) (Genscript) using PCR-based QuickChange Site-directed Mutagenesis Kit (Agilent Technologies**,** #200518) according to the manufacturer’s protocol. Cycling parameters were 95°C for 30 s., 70°C for 60 s., and 68°C for 14 min at a total of 30 cycles. Forward primer: 5′-cgtgggcccaggagaggggccca-3′. Reverse primer: 5′-tgggcccctctcctgggcccacg-3′. Correct introduction of A27G mutation was confirmed with sequencing (forward and reverse strand) (Eurofins).

Cells were plated onto 96-well solid white tissue culture plates for assaying (15,000 cells/well) one day prior to transient co-transfection using calcium phosphate co-precipitation. In brief, DNA (receptor:CAMYEL DNA ratio 1:5) mixed with CaCl_2_ (2 M) and TE-buffer (10 mM Tris-HCl, 1 mM EDTA, pH 7.5) was dropwise added to 2XHBS (50 mM HEPES, 280 mM NaCl, 1.5 mM NaH_2_PO_4_, pH 7.2) and incubated for 45 min RT. The mixture and a final concentration of 100 μM Chloroquine (Sigma-Aldrich, C6628) were added to the cells and left to incubate for 5 h at 37°C before replacement of the growth media. BRET-based cAMP assay was performed 24-48 hours after transfection: Cells were washed twice with Hank’s balanced salt solution (HBSS) (Gibco, #14025092) and incubated in HBSS, pH 7.4 for 30 min at 37°C prior to BRET measurements. BRET measurements were carried out using CLARIOstar Plus plate reader (BMG LabTech). Emission signals from Renilla luciferase and YFP were measured simultaneously using a BRET filter set (475-30/535-30). Cells were assayed in a total of 100 μL HBSS containing 5 μM coelenterazine h (Invitrogen, C6780) for 30-60 min with temperature set at 37°C. BRET signal was calculated as a ratio (535/475) of emission signals and normalized to t=0 s. cAMP production as presented in arbitrary units (AU) was calculated by multiplying all BRET-values by -1. The presented data represent an average of 3 independent biological replicas.

#### Bioluminescence resonance energy transfer (BRET)-based miniG subtype recruitment

G protein-coupling was assessed using a BRET-based miniG subtype recruitment assay applying NES-NanoLuc-MiniG subtypes (miniGi, miniGs, miniGq, miniG12) and a plasma membrane marker Venus-Kras in live HEK293-T cells (ATCC).

Cells were seeded onto a 6-well tissue culture plate the day prior to transfection to achieve 70-80% confluence. DNA transfection was performed according to the manufacturer’s protocol (Lipofectamine 2000). In brief, per well 100 μL of Opti-MEM (Gibco, 31985) was mixed with 3 μL of Lipofectamine 2000 (Invitrogen, 11668019) and incubated at RT for 5 min. Increasing amounts of *GPR3* coding sequence (NM_005281.4) in a custom pcDNA3.1(+) (Genscript) or DRY-mutant *GPR3* pcDNA3.1(+) (Genscript) (0 ng, 50 ng, 100 ng, 250 ng, 500 ng) was added together with 50 ng DNA encoding NES-NanoLuc-MiniG subtypes (miniGi, miniGs, miniGq, miniG12) and 500 ng plasma membrane marker Venus-Kras (both provided by Nevin Lambert, Augusta University, GA, USA). The transfection mix incubated at RT for 20 min to enable lipid:DNA complexes to form.

Subsequently, the DNA/lipid mix was added directly onto the cells and left to incubate O/N. 24h post transfection, cells were washed with PBS and resuspended in FluoroBrite DMEM phenol red-free media (Gibco, A1896701) supplemented with 5% FBS (Gibco, 10500-064-500) and 10% L-Glutamine and distributed onto a poly-D-lysine-coated white 96-well plate at 100,000 cells/well. 48 h post transfection, media was replaced with Hanks’ balanced salt solution (Gibco, 24020117) supplemented with Nano-Glo (Promega, #N1110) in 1:500 dilution. BRET measurements were recorded over an 8 min period (times points: 0, 2, 4, 6, 8 min) using a PHERAstar Microplate Reader (BMG Labtech) with BMG BRET1 filters: Donor wavelength: 475-30 and acceptor wavelength: 535-30 at 37°C. The BRET ratio (acceptor/donor) for each time point was individually calculated and averaged. The BRET signal measuring miniG translocation to the plasma membrane was normalized by subtracting an averaged background BRET ratio from all measurements.

#### Enzyme fragment complementation (EFC)-based cAMP sensing

cAMP production in COS-7 cells transiently transfected with increasing amounts of *GPR3* was investigated using the HitHunter® cAMP Assay for Small Molecules (Eurofins DiscoverX, #90-0075SM10). COS-7 cells were plated onto 96-well solid white tissue culture plates for assaying (20,000 cells/well) one day prior to transient transfection with increasing amounts of the human *GPR3* coding sequence (NM_005281.4) in a custom pcDNA3.1(+) (Genscript) (2.5 μg, 10 μg, 20 μg, 30 μg, 40 μg, 50 μg) or EV control (60 μg). Transient transfection was performed as previously described for BRET-based cAMP sensing. HitHunter® cAMP Assay for Small Molecules was performed according to the manufacturer’s protocol 24h after transfection. cAMP assay buffer was HEPES buffered saline (HBS) with 1 mM (final) 3-Isobutyl-1-methylxanthine (IBMX) (Sigma-Aldrich, I5879). Luminescence was read on EnVision Multilabel plate reader (PerkinElmer). cAMP measurements were fitted using Sigmoidal, 4PL, X is log(concentration) fit line calculated with GraphPad Prism software. Gene expression was measured by real-time (RT) qPCR as described above and normalized to EV. Presented data were fitted using Sigmoidal, 4PL, X is concentration fit line calculated with GraphPad Prism software (x-axis on log10 scale). The presented data represent an average of 3 independent biological replicas.

#### *GPR3* truncation

Stepwise N-terminal truncation was performed in the *GPR3* coding sequence (NM_005281.4) in a custom pcDNA3.1(+) (Genscript) using PCR-based QuickChange Site-directed Mutagenesis Kit (Agilent Technologies**,** #200518) according to the manufacturer’s protocol. Cycling parameters were 95°C for 30 s., 70°C for 60 s., and 68°C for 14 min at a total of 30 cycles. All truncations begin with and include amino acid position 2 and extend increasingly further into *GPR3* N-terminus, thus leaving the start codon (position 1, Methionine). The amino acid positions removed by truncation are indicated for each primer set. A complete list of applied primers is available in the KRT. Truncations were confirmed with sequencing (forward and reverse strand) (Eurofins). cAMP production from truncated *GPR3* versions was investigated using transient transfection and BRET-based cAMP sensing assay as described above. cAMP production as presented in arbitrary units (AU) was calculated by multiplying all BRET-values by -1, and the cAMP production induced by WT *GPR3* was set to 100%.

#### GPR3 N-terminal peptide titration

A peptide corresponding to GPR3 amino acid positions 18 through 27 (see KRT for the full sequence) was acquired from peptides&elephants and dissolved in DMSO. The potential of the peptide fragment to dose-dependently activate GPR3 or Cannabinoid receptor type 1 (CB1) was evaluated with the BRET-based cAMP sensing assay as described above using plasmids carrying either *GPR3* coding sequence (NM_005281.4) or *CB1* coding sequence (NM_016083.6) in a custom pcDNA3.1(+) (Genscript).

The background signal was subtracted from all measurements, before the BRET(535/475) ratio induced by addition of a vehicle (DMSO) was subtracted from the BRET(535/475) ratio induced by the peptide. Presented net AUCs were calculated over the time-course of 20 min. cAMP production as presented in arbitrary units (AU) was calculated by multiplying all AUC-values by -1 and adding +1. cAMP measurements were fitted using Sigmoidal, 4PL, X is log(concentration) fit line calculated with GraphPad Prism software. The presented data represent an average of 3 independent biological replicas.

### Quantification and statistical analysis

All statistical tests were performed using GraphPad Prism software. Data are presented as means+SEM unless otherwise stated. Box plots are presented as box: 25^th^ to 75^th^ percentile and whiskers: Min to max. Data with two groups were analyzed using unpaired two-tailed student’s t-test. Data with two paired groups were analyzed using paired two-tailed student’s t-test. Data with two groups and repeated measurements were analyzed using two-way ANOVA with Bonferroni’s correction. Data comparing multiple groups to a single control group were analyzed using Bonferroni’s multiple comparison test and comparing each cell mean with the control cell mean. RNA-sequencing data and qPCR array data were analyzed as stated in the designated paragraph. Significance is indicated as follows: p≤0.05=^∗^, p≤0.01=^∗∗^, p≤0.001=^∗∗∗^, p≤0.0001=^∗∗∗∗^.

Presented histology images are representative of biological replicas. In *in vivo* studies, n (individually stated for each experiment) represents the number of animals in each group. Unless otherwise stated, the *in vitro* data presented in the article represent a single representative biological replicate. Each *in vitro* study was repeated 2-5 times. Thus, error bars indicate the technical variance in each experiment.
