# Supplementary material for: Lipolysis drives expression of the constitutively active receptor GPR3 to induce adipose thermogenesis
Source: Cell. 2021 Jun 24;184(13):3502–3518.e33. doi: 10.1016/j.cell.2021.04.037 (PMC8238500; doi:10.1016/j.cell.2021.04.037)
Supplement: Document S1. Tables S1–S3 [file mmc1.pdf]

Cell, Volume *184*

Supplemental information

Lipolysis drives expression

# of the constitutively active receptor GPR3

to induce adipose thermogenesis

Olivia Sveidahl Johansen, Tao Ma, Jakob Bondo Hansen, Lasse Kruse Markussen, Renate Schreiber, Laia Reverte-Salisa, Hua Dong, Dan Ploug Christensen, Wenfei Sun, Thorsten Gnad, Iuliia Karavaeva, Thomas Svava Nielsen, Sander Kooijman, Cheryl Cero, Oksana Dmytriyeva, Yachen Shen, Maria Razzoli, Shannon L. O'Brien, Eline N. Kuipers, Carsten Haagen Nielsen, William Orchard, Nienke Willemsen, Naja Zenius Jespersen, Morten Lundh, Elahu Gosney Sustarsic, Cecilie Mørch Hallgren, Mikkel Frost, Seth McGonigle, Marie Sophie Isidor, Christa Broholm, Oluf Pedersen, Jacob Bo Hansen, Niels Grarup, Torben Hansen, Andreas Kjær, James G. Granneman, M. Madan Babu, Davide Calebiro, Søren Nielsen, Mikael Rydén, Raymond Soccio, Patrick C.N. Rensen, Jonas Thue Treebak, Thue Walter Schwartz, Brice Emanuelli, Alessandro Bartolomucci, Alexander Pfeifer, Rudolf Zechner, Camilla Scheele, Susanne Mandrup, and Zachary Gerhart-Hines

Table S1. Relative cold induction of Gs-coupled GPCRs in BAT, Related to Figure 1.

Receptor	TN	3 hours cold	8 hours cold	24 hours cold	3 days cold	1 week cold
Adcyap1r1	1	0.886	0.789	1.226	1.320	1.083
Adora2a	1	1.218	1.367	1.475	0.677	0.676
Adora2b	1	0.642	0.635	0.700	0.779	1.119
Adrb1	1	1.349	1.297	1.164	1.399	0.910
Adrb2	1	0.279	0.316	0.173	0.125	0.137
Adrb3	1	0.202	0.425	0.440	1.509	1.003
Avpr2	1	1.024	0.734	1.629	0.817	2.172
Calcrl	1	0.617	1.063	1.537	1.089	1.598
Crhr1	1	0.559	0.817	1.594	1.208	1.414
Crhr2	1	0.951	1.255	2.091	1.599	2.407
Drd1a	1	2.673	13.384 [****]	10.043 [***]	4.394	18.786 [****]
Drd5	1	0.292	1.109	2.381	3.532	1.556
Fshr	1	1.999	1.157	1.478	1.508	1.495
Gcgr	1	1.014	1.325	1.313	0.515	1.182
Ghrhr	1	0.908	0.873	6.309	2.935	2.733
Gipr	1	0.526	0.538	0.763	1.057	0.479
Glp1r	1	1.359	1.795	1.562	1.375	1.426
Glp2r	1	2.905	1.897	1.654	1.175	2.377
Gpbar1	1	1.554	0.838	0.654	1.100	0.622
Gpr101	1	0.301	1.456	0.254	1.710	0.541
Gpr119	1	1.954	2.432	1.579	1.122	7.595 [*]
Gpr133	1	2.170	1.409	1.853	1.921	2.820
Gpr26	1	1.165	1.160	1.483	2.547	1.329
Gpr3	1	30.042 [****]	63.521[****]	67.077 [****]	52.432 [****]	87.234 [****]
Gpr65	1	0.756	1.030	0.639	0.780	2.103
Htr7	1	0.675	0.846	0.604	0.898	1.070
Lhcgr	1	0.360	0.957	5.239	4.490	1.262
Mc1r	1	1.134	0.843	0.772	0.281	0.532
Mc2r	1	0.151	0.192	0.290	0.247	0.175
Mc3r	1	1.275	1.431	1.581	1.325	1.562
Mc4r	1	1.597	2.215	1.234	3.078	1.497
Mc5r	1	0.998	1.185	0.436	1.062	1.227
Ptgdr	1	2.598	1.458	3.189	2.545	1.350
Ptger2	1	1.079	0.970	2.589	0.325	2.045
Ptger4	1	0.697	0.910	0.902	0.354	0.812
Ptgir	1	0.685	0.984	0.737	0.731	1.350
Pth1r	1	0.414	0.707	0.725	0.667	0.492
Rxfp2	1	0.821	1.584	1.191	2.346	1.014
Sctr	1	0.649	0.265	0.113	0.252	0.252
Taar1	1	1.360	0.886	0.840	0.807	1.313
Taar4	1	2.854	2.207	2.455	2.444	1.569
Tshr	1	0.690	0.687	0.878	1.508	0.787
Vipr1	1	2.488	0.519	0.821	1.583	0.764
Vipr2	1	0.921	0.607	0.508	0.892	0.620

Table S2. Relative cold induction of Gs-coupled GPCRs in scWAT, Related to Figure 1.

Receptor	TN	1 week cold
Adcyap1r1	1	1.12435
Adora2a	1	1.015275
Adora2b	1	0.759228
Adrb1	1	2.319545 [*]
Adrb2	1	0.490304
Adrb3	1	0.877347
Avpr2	1	0.698586
Calcrl	1	0.74712
Crhr1	1	1.026174
Crhr2	1	0.551537
Drd1a	1	2.073623
Drd5	1	0.928179
Fshr	1	0.957577
Gcgr	1	0.517928
Ghrhr	1	1.421452
Gipr	1	0.552729
Glp1r	1	0.364139
Glp2r	1	0.593368
Gpbar1	1	0.706243
Gpr101	1	0.967332
Gpr119	1	0.942241
Gpr133	1	0.636265
Gpr26	1	0.548419
Gpr3	1	4.684511 [****]
Gpr65	1	0.660672
Htr7	1	1.04161
Lhcgr	1	1.09412
Mc1r	1	0.542785
Mc2r	1	0.570388
Mc3r	1	0.727088
Mc4r	1	1.197985
Mc5r	1	1.57169
Ptgdr	1	1.083403
Ptger2	1	0.405265
Ptger4	1	0.512577
Ptgir	1	0.742936
Pth1r	1	0.609481
Rxfp2	1	1.222464
Sctr	1	1.02965
Taar1	1	0.850346
Taar4	1	0.627048
Tshr	1	0.691152
Vipr1	1	0.593946
Vipr2	1	1.454639

Table S3. List of primers, Related to STAR Methods.

Transcript	Forward primer	Reverse primer
Gpr3 (ORF)	ATGTTCCTGCTGGTGGGTAG	GTGAGCCAATGCAGAAGTCA
Gpr3 (UTR)	ATCACCTGAGCAACCGAGAA	AGATGGGGGTGCATTTTACA
36b4	TCATCCAGCAGGTGTTTGACA	GGCACCGAGGCAACAGTT
Ucp1	GGATTGGCCTCTACGACTCA	TAAGCCGGCTGAGATCTTGT
Bmp8b	AGCCTGACTTGAACCTGGAA	ACCCCCAAGAGACAGGCTAT
Dio2	GCTTCCTCCTAGATGCCTACAA	AGTCAAGAAGGTGGCATTCG
Lpl	CCCTAAGGACCCCTGAAGAC	GGCCCGATACAACCAGTCTA
Acadm	GCCCAGAGAGCTCTAGACGA	GCGAGCAGAAATGAAACTCC
Elovl3	ATGAATTTCTCACGCGGGTT	AGCTTACCCAGTACTCCTCCA
Adcy3	GGAAAAGGACTCTCCTATGGTGG	CTGTCAGTGCCATTGAGCCC
Adrb1	CTACAACGACCCCAAGTGCT	GGCACGTAGAAGGAGACGAC
Adrb2	CAGGAACTGCTGTGTGAGGA	CTTGGGAGTCAACGCTAAGG
Adrb3	AGGAAGCTTGCTTGATCCCC	GCATCCATAGCCGTTGCTTG
Pparg	AGAACCTGCATCTCCACCTT	CCGGCAGTTAAGATCACACC
Ppara	AATTTGCTGTGGAGATCGGC	TTAAGCACGTGCACAATCCC
Srebp1	CTTACCCCTCCACCCTCAGA	TGTCGGGCTCAGAGTCACTA
Lxra	TCGTGTCCGTGCAGGAGATT	TCTCCAGAAGCATGACCTCGAT
Lxrb	CCCCACCCATTGAGTCTTCC	CTCCACTCAAGGTGCATGGT
GPR3	GTCCTGAGCGGTACCATGA	CCCACGCTGCTTACATTCAC
36B4	TTTGTGTTCACCAAGGAGGA	GTGACTTCACATGGGGCAAT
UCP1	AAGGCTTGACGGGTCTTTG	CGATAAGAGCCGACACCAAG
PRDM16	CGGCAAAGGAGACAGACTTC	CATCCACGCAGAACTTCTCA
FABP4	TACTGGGCCAGGAATTTGAC	TACCAGGACACCCCCATCTA
DIO2	GGAAGAGCTTCCTCCTCGAT	CTGGAGACATGCACCACACT
GLUT4	AACTGGACGAGCAACTTCATC	AGGACCGCAAATAGAAGGAAG
NDUFA	CCGAACACTGTGCACCAATTC	GGGTCTGTGACATGGGCATT
COX7A1	CCGCTTTCAGAACCGAGTG	AGAGGCCAGCGTTTATTGACA
CRLS1	GCTTGGCCCCAGTTCTGG	GGCCCAGTTTCGAGCAATAA
GPD2	GAAGTGGCTGTGCGCTAGAT	TAGTGCTTCTGCTGCTGGTC
FASN	AAGGACCTGTCTAGGTTTGATGC	TGGCTTCATAGGTGACTTCCA
ADIPOQ	CCTGGTGAGAAGGGTGAGAA	CTCCTTTCCTGCCTTGGATT
